# SPSB1 Promotes Subcutaneous Adipose Hyperplasia in Facial Port‐Wine Stains by Controlling HDAC1 Degradation and Stability Through Two Distinct Proteolytic Pathways

**DOI:** 10.1002/advs.76699

**Published:** 2026-07-20

**Authors:** Hongrui Chen, Bin Sun, Yajing Qiu, Chen Hua, Xiaoxi Lin, Wei Gao

**Affiliations:** ^1^ Department of Plastic & Reconstructive Medicine Shanghai Ninth People's Hospital Shanghai Jiao Tong University School of Medicine Shanghai China; ^2^ Department of Laser and Aesthetic Medicine Shanghai Ninth People's Hospital Shanghai Jiao Tong University School of Medicine Shanghai China

**Keywords:** adipogenesis, adipose tissue, cell biology, chromatin, progenitor cell, ubiquitin ligase

## Abstract

Facial port‐wine stains (PWS) are often complicated by subcutaneous adipose hyperplasia, yet the underlying cellular and molecular mechanisms remain unclear. Here, using single‐cell RNA sequencing of facial adipose tissues, we identified a distinct subpopulation of adipose stem and progenitor cells that expressed high levels of SPSB1 in hypertrophic PWS. SPSB1 expression was transcriptionally upregulated by FOSL1. Functional assays revealed that SPSB1 potently promoted adipogenesis both in vitro and in vivo. Mechanistically, SPSB1, acting as an adapter for the Cullin‐RING E3 ligase complex, directly interacted with HDAC1. It specifically mediated K48‐ and K29‐linked polyubiquitination of HDAC1 at lysine residues 89 and 361, respectively, thereby targeting HDAC1 for degradation via both the ubiquitin‐proteasome and autophagy‐lysosome pathways. The degradation of HDAC1 increased chromatin accessibility of key adipogenic genes and promoted their expression. Furthermore, HDAC1 downregulation relieved its transcriptional repression on the SPSB1 promoter, establishing a positive feedback loop that perpetuated the pro‐adipogenic signal. Collectively, our findings delineated a novel SPSB1‐HDAC1 regulatory axis that drove pathological adipose hyperplasia in facial PWS, highlighting SPSB1 and HDAC1 as potential therapeutic targets for this condition.

## Graphical abstract

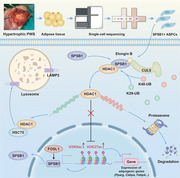

## 1 Introduction

Facial port‐wine stain (PWS) is a congenital, progressive capillary malformation typically presenting as red to purple skin patches. Among its subtypes, the hypertrophic form represents an advanced stage, characterized not only by the dilation and proliferation of dermal vessels but also by significant soft‐tissue hypertrophy in the subcutaneous layer, particularly involving abnormal adipose tissue hyperplasia [1]. Adipose hyperplasia can lead to facial contour alterations, asymmetry, and functional impairment, making it a primary concern for patients seeking treatment [2]. However, the cellular and molecular mechanisms underlying aberrant subcutaneous fat expansion in hypertrophic PWS remain poorly understood. Recent advancements in technologies such as single‐cell transcriptomics have shifted research focus toward the heterogeneity of adipose‐derived stem and progenitor cells (ASPCs) within the adipose stroma and their role in tissue dysregulation. For example, in cachexia, ASPCs exhibit a marked pro‐inflammatory shift accompanied by significantly remodeled interactions with immune cells [3]. PRG4+/CLEC3B+ ASPCs are notably expanded in lymphedema and are associated with adipose tissue fibrosis [4]. Elucidating the key cellular populations and molecular pathways driving subcutaneous adipose hyperplasia is crucial for understanding pathogenesis of PWS.

Post‐translational modifications (PTMs), such as ubiquitination, SUMOylation, acetylation, and methylation, have been widely implicated in regulating adipogenesis [5, 6, 7, 8]. Among these, ubiquitination has been extensively studied. Ubiquitin is a 76‐amino acid polypeptide containing seven lysine residues (K6, K11, K27, K29, K33, K48, and K63) that can be conjugated to substrate proteins [9]. Certain molecules control adipogenesis by directly modulating polyubiquitin chains linked via specific lysine residues on key adipogenic proteins. For example, USP10 directly interacts with C/EBPβ and stabilizes it through deubiquitination [10]. Ubiquitination also influences lipogenesis by regulating the degradation of upstream signaling proteins. HERP interacts with STEAP4 to prevent its ubiquitin‐mediated degradation, thereby enhancing adipogenesis in a PPARγ‐dependent manner [11]. Similarly, ANXA1 promotes SMAD4 ubiquitination and degradation, which reduces PPARγ transcription and thereby inhibiting adipogenesis [12]. Following ubiquitination catalyzed by E3 ligases, the ubiquitin‐proteasome system (UPS) or the autophagy‐lysosome pathway (ALP) recognizes and degrades the modified substrate proteins. These represent two major pathways for maintaining protein homeostasis in eukaryotes. Accumulating evidence indicates that both the UPS and ALP are critically involved in regulating lipid metabolism and may mutually affect each other during adipogenesis [13]. Nevertheless, the specific roles of the UPS and ALP in subcutaneous adipose hyperplasia of PWS have not been elucidated.

Cullin‐RING E3 ubiquitin ligases (CRLs) function as multi‐protein complexes in which a Cullin protein serves as a scaffold to recruit specific adaptor proteins. These adaptors, in turn, recognize and facilitate the ubiquitination of specific substrate proteins [14]. SPSB1 (SPRY domain‐containing SOCS box protein 1) is a member of the SPSB protein family. Proteins in this family typically contain an N‐terminal SPRY domain and a C‐terminal SOCS box. The SPRY domain mediates specific protein‐protein interactions, while the SOCS box recruits components such as Elongin B/C and Cullin 5 to form the ECS (Elongin B/C‐Cul5‐SOCS‐box protein) complex. Acting as an adaptor, SPSB1 thereby targets specific substrates for ubiquitination, regulating their stability, localization, and function [15, 16, 17]. SPSB1 has been implicated in various physiological and pathological processes. In immune regulation, it can modulate innate immune responses by mediating the ubiquitination and degradation of signaling proteins such as NF‐κB [18]. SPSB1‐mediated ubiquitination of HnRNP A1 also regulates alternative splicing and cell migration after the EGF stimulation [19]. However, the precise role of SPSB1 in pathological adipose hyperplasia has not been systematically investigated.

This study provides a comprehensive, single‐cell level analysis of ASPC function within the subcutaneous adipose tissue of hypertrophic PWS. We identified a distinct subpopulation of ASPCs characterized by high SPSB1 expression (SPSB+ ASPCs), which exhibited a markedly enhanced capacity for adipogenesis. Through trajectory analysis, chromatin immunoprecipitation (ChIP)‐qPCR, and luciferase reporter assays, we demonstrated that the transcription factor FOSL1 drove the formation of this SPSB+ ASPC population. Mechanistically, SPSB1 catalyzed K48‐ and K29‐linked polyubiquitination of HDAC1. This modification targeted HDAC1 for degradation via dual proteolytic pathways‐both the UPS and the ALP. HDAC1 degradation increased the abundance of H3K27ac and H3K9ac at the promoters of key adipogenic genes, promoting chromatin opening and gene transcription and forming a positive feedback loop that facilitates adipogenesis. This work offers novel insights into the cooperative roles of the UPS and ALP in adipogenesis and demonstrates that a Cullin5 adaptor protein SPSB1 can simultaneously mediate substrate degradation through both proteasomal and lysosomal pathways.

## 2 Results

### 2.1 Single‐Cell Sequencing Revealed Cellular Diversity and Heterogeneity in Facial Adipose Tissue of Hypertrophic PWS

**FIGURE 1:**
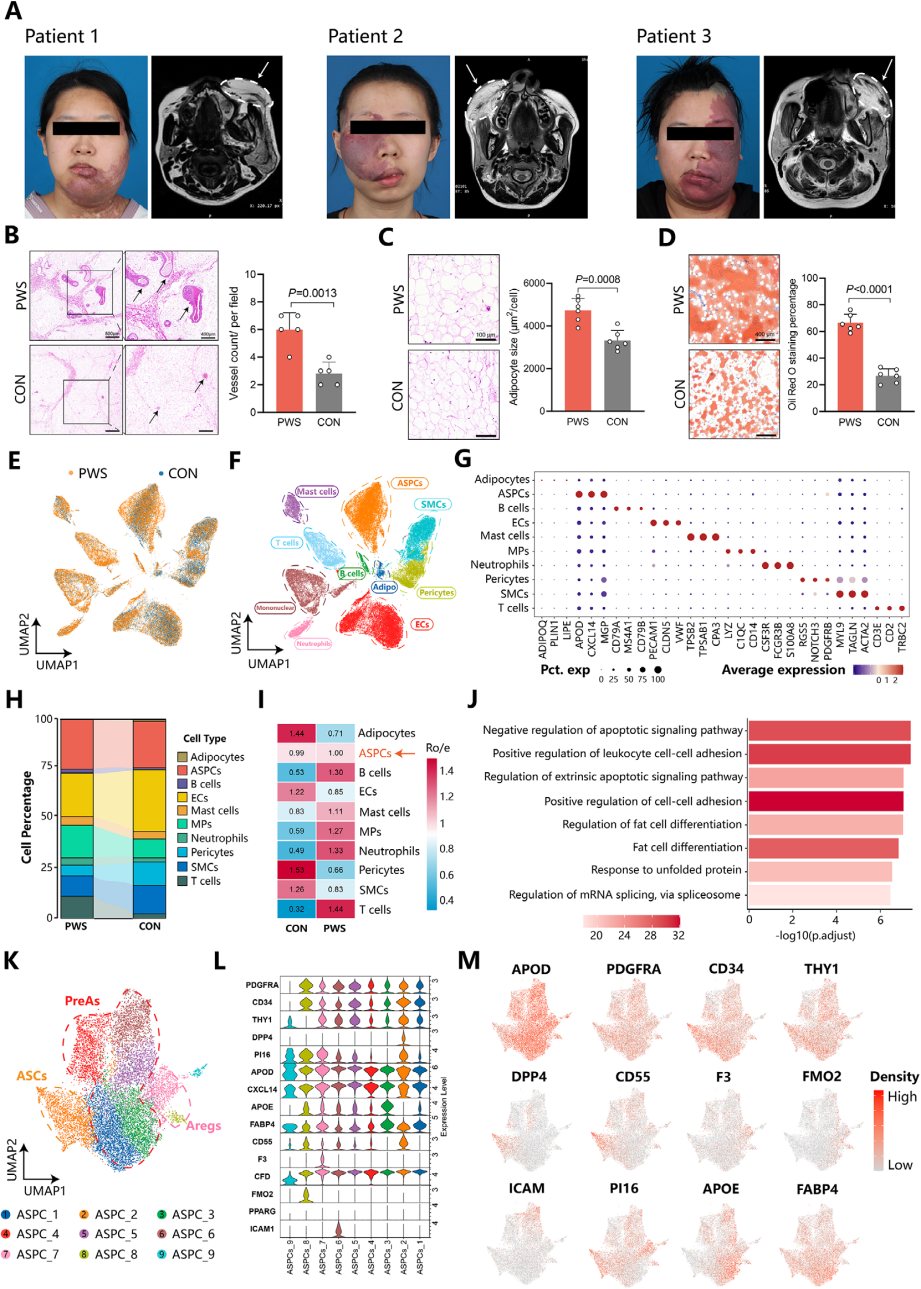
Landscape of cells in the SVF of adipose tissues from patients with or without hypertrophic PWS. (A): Clinical and radiological manifestations of PWS patients. (B): Histological characteristics of subcutaneous adipose tissue in PWS. Black arrows indicate blood vessels within the adipose tissue. Scale bar: 800 µm (left) and 400 µm (right). (C): H&E staining of adipose tissue showed adipocyte size. Scale bar: 100 µm. (D): Oil Red O staining and quantitative analysis of the proportion of the Oil Red O staining area within the whole visual field of adipose tissue from the PWS and CON (representing the area of the lipid droplets). Scale bar: 400 µm. (E): Uniform Manifold Approximation and Projection (UMAP) plot showing the origins of cells. (F): UMAP plot of single cells showing 10 major cell types by manual annotation. (G): Bubble heatmap showing expression levels of selected marker genes for each cell type. (H): Sankey diagram showing proportions of identified major cell populations within adipose tissue. (I): Heatmap showing the Ro/e index of ASPCs. (J): Metascape showing the enriched pathways within ASPCs from PWS based on upregulated genes in this cell population. (K): UMAP visualization of inferred ASPCs from adipose tissue identified nine ASPCs subpopulations. (L): Violin plot showing the expression of marker genes within each cell subpopulations. (M): Distribution map of the selected genes expressed in different subgroups. The color depth represents the intensity of expression in the sample. The data were presented as the mean±SD from at least five different samples (B, C, D).

We first collected facial adipose tissue from patients with PWS who underwent surgery. All patients presented with typical proliferative PWS and marked facial hypertrophy. Magnetic resonance imaging confirmed hyperplasia of adipose tissue on the affected maxillofacial side (Figure 1A). Considering the potential heterogeneity of adipose tissue from different regions [15], adipose samples from patients who underwent surgery for other facial skin lesions were used as controls. Hematoxylin and eosin (H&E) staining revealed that, compared with normal facial adipose tissue, the subcutaneous adipose tissue of PWS contained more small blood vessels with more dilated lumens (Figure 1B). Moreover, adipocytes in the PWS group were significantly enlarged compared with those in the control group (Figure 1C). Oil Red O staining demonstrated increased lipid accumulation in the adipose tissue of the PWS group (Figure 1D).

To accurately delineate the cellular heterogeneity of facial adipose tissue in PWS, we isolated the stromal vascular fraction (SVF) from facial fat samples of three PWS patients and performed single‑cell RNA sequencing (GSA‐Human: HRA015590). After stringent quality control, transcriptomic data from 48,341 cells were obtained for subsequent comparative analysis with our previously published single‑cell sequencing dataset of normal facial adipose tissue (GEO: GSE267777) (Figure 1E). Unbiased clustering identified 10 major cell lineages (Figure 1F), with representative markers for each lineage shown in Figure 1G. Comparison of cell‑type proportions revealed that the percentage of ASPCs was similar between the PWS and control groups, as reflected by a comparable Ro/e index (Figure 1H,I). Several genes, including Cebpa, Cebpb and Klf4, were significantly elevated in ASPCs from the PWS group and were known to promote adipogenesis (Figure S1A). Enrichment analysis further indicated that pathways related to fat cell differentiation were significantly upregulated in ASPCs from the PWS group (Figure 1J).

Given the crucial role of ASPCs in adipose expansion and regeneration, we further explored their heterogeneity to identify distinct subpopulations. As previously reported, the adipose progenitor lineage can be divided into three main populations: adipose stem cells (ASCs), which exhibit stem‐like properties and multipotent differentiation capacity; preadipocytes (PreAs), a group committed to adipogenesis; and adipogenesis‐regulatory cells (Aregs), which inhibit the adipogenesis of other precursor cells [20, 21]. Accordingly, we analyzed the potential functional states of the annotated cell clusters in our samples. We identified nine subclusters (Figure 1K). Cluster 2 showed high expression of canonical ASC markers such as DPP4, CD55, and Pi16 and was annotated as ASCs, representing the most pluripotent stem cell population [22]. Clusters 7 and 8 expressed elevated levels of FMO2 and F3 (CD142), markers characteristic of Aregs [21]. The remaining clusters were characterized by high expression of early adipogenic genes such as FABP4, APOD, and APOE, yet notably lacked expression of PPARG, the master regulator of adipocyte differentiation and a defining marker of committed preadipocytes (Figure 1L,M). As described by Siersbæk et al., adipocyte differentiation proceeds through sequential waves of transcription factor activation, wherein PPARG induction occurs at a later stage following the initial establishment of early adipogenic competence [23]. The absence of PPARG expression across all nine subclusters therefore reflected these cells represented an early PreAs stage.

### 2.2 SPSB1^+^ ASPCs Represented a Unique Subpopulation in the Adipose Tissue of Hypertrophic PWS

**FIGURE 2:**
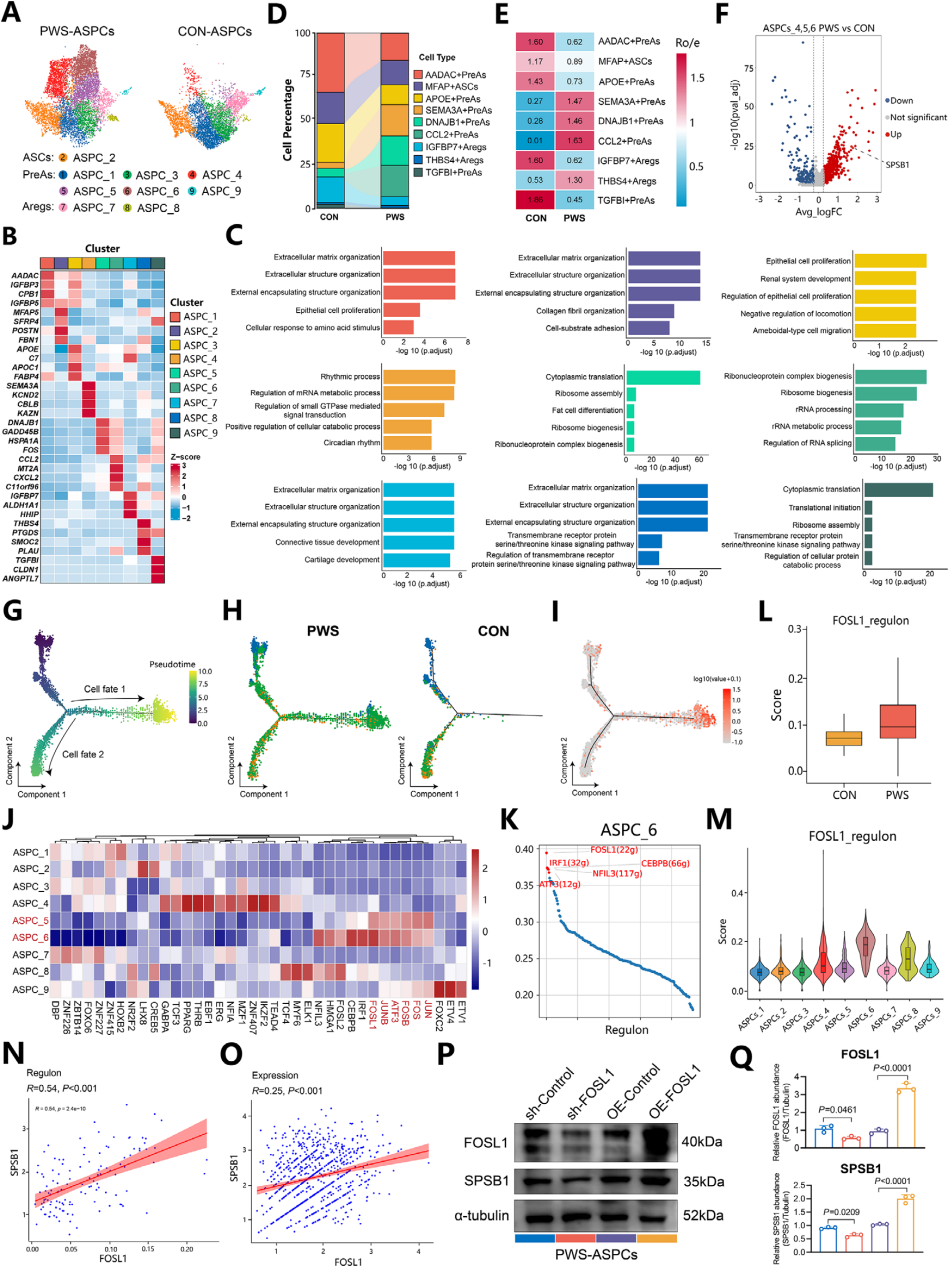
SPSB^+^ ASPCs was a unique subpopulation in the adipose tissue of PWS. (A): Focusing on all ASPCs, dimensionality reduction and unsupervised clustering depicting the mapping of nine cell subpopulations of ASPCs. (B): Heatmap illustrating highly expressed genes among identified nine ASPCs subpopulations. (C): Functional enrichment analysis uncovered specific biological pathways among different ASPCs subpopulations. (D, E): Sankey diagram displaying proportions and Ro/e index supporting the preference of identified nine ASPCs subpopulations. (F): Volcano plot showing the differences in the expression of genes in PWS‐ASPCs‐4, 5, 6 and CON‐ASPCs‐4, 5, 6. (G, H): Pseudo‐time trajectory map of ASPCs in PWS and CON classified by manual annotation. (I): Pseudo‐time trajectory map showed the SPSB1 expression. (J): SCENIC analysis evaluated transcription factor activity across distinct ASPCs subpopulations. (K): Regulon specificity score (RSS) plot displaying FOSL1 as the top‐ranked transcription factor in SPSB^+^ ASPCs. (L): Box plot comparing the RSS levels of FOS between ASPCs derived from PWS and CON. (M): Violin plot comparing the RSS levels of FOSL1 across ASPCs subpopulations. (N, O): The correlation of FOSL1 expression and transcriptional activity with SPSB1 expression. (P, Q): Western blot (WB) and quantitative analysis of SPSB1 protein level in PWS‐ASPCs with FOSL1 knockdown or overexpression. Data were analyzed by unpaired two‐sided Student's t tests (Q) and were presented as mean ± SD with three replicate experiments. Original blot can be found in Figure S10.

To elucidate the role of ASPCs in adipose tissue expansion, we further classified them into nine subpopulations based on distinct expression profiles and functional characteristics (Figure 2A–C). For example, ASPCs‐1 and ASPCs‐2 were enriched in extracellular matrix organization, consistent with their identity requiring ECM remodeling for adipogenesis [24]. ASPCs‐3 showed epithelial cell proliferation, suggesting a progenitor‐like state [25]. ASPCs‐4 displayed rhythmic processes and circadian rhythm, aligning with the known circadian regulation of adipose tissue metabolism [26]. ASPCs‐5 directly enriched for fat cell differentiation. Among these subpopulations, the proportions of SEMA3A^+^ PreAs (ASPCs‑4), DNAJB1^+^ PreAs (ASPCs‑5), and CCL2^+^ PreAs (ASPCs‑6) were significantly increased in the PWS group, suggesting a potential link to subcutaneous fat accumulation (Figure 2D–E). Notably, expression of SPSB1 was markedly elevated in ASPCs‑4, ‑5, and ‑6 (Figure 2F). Pseudotemporal trajectory analysis revealed two distinct differentiation fates among ASPCs (Figure 2G). Cells from the PWS cohort exhibited a stronger bias toward differentiation fate 1 (Figure 2H), and SPSB1 expression progressively increased along this trajectory (Figure 2I). Differentiation potential inferred by CytoTRACE indicated that ASPCs‑5 and ASPCs‑6 retained certain differentiation capacity, while ASPCs‑4 appeared more mature and functionally committed (Figure S1B,C) [27]. Based on these findings, we focused our subsequent investigations on clusters 5 and 6.

To identify the transcription factors driving the specific upregulation of SPSB1, we employed the SCENIC pipeline to elucidate the transcriptional regulatory networks across different ASPC subpopulations and pinpoint key transcription factors specific to each cell cluster (Figure 2J) [28]. Within subclusters 5 and 6, FOSL1 was identified as a potential transcriptional regulator based on its highest regulon specificity score (Figure 2K). Furthermore, FOSL1 exhibited an elevated regulon activity score in PWS‑ASPCs, suggesting increased activity under pathological conditions, which likely led to broader activation of downstream target genes (Figure 2L,M). Correlation analysis showed that SPSB1 expression was significantly associated with both the activity and expression level of the FOSL1 (Figure 2N,O), indicating that FOSL1 may regulate SPSB1 expression.

To validate the regulatory role of FOSL1 on SPSB1, we performed ChIP‐qPCR, which demonstrated significant enrichment of FOSL1 at the promoter region of SPSB1 (Figure S2A). In addition, luciferase reporter assays showed that FOSL1 markedly enhanced the activity of the wild‑type SPSB1 promoter (Figure S2B). To further confirm that FOSL1 upregulated SPSB1 expression, we conducted qPCR and found that knockdown of FOSL1 reduced SPSB1 mRNA levels, while FOSL1 overexpression increased them (Figure S2C,D). We further examined protein expression in PWS‑ASPCs and in an immortalized ASPC cell line derived from facial infiltrating lipomatosis (Im‑FIL‑ASPCs). Facial infiltrating lipomatosis (FIL) closely resembles the subcutaneous fat hyperplasia seen in hypertrophic PWS, both exhibiting marked adipose expansion [29]. Our previous data have also established Im FIL‐ASPCs as a robust tool for studying adipogenesis [30]. Consequently, the parallel use of PWS‐ASPCs and Im FIL‐ASPCs provides a powerful strategy for validating gene function. The results showed that FOSL1 knockdown decreased SPSB1 protein expression, whereas FOSL1 overexpression had the opposite effect (Figure 2P,Q, Figure S2E,F). Together, these findings demonstrated an important role for FOSL1 in promoting SPSB1 expression in PWS‑ASPCs.

### 2.3 SPSB1 Promoted Adipogenesis both in Vitro and in Vivo

**FIGURE 3:**
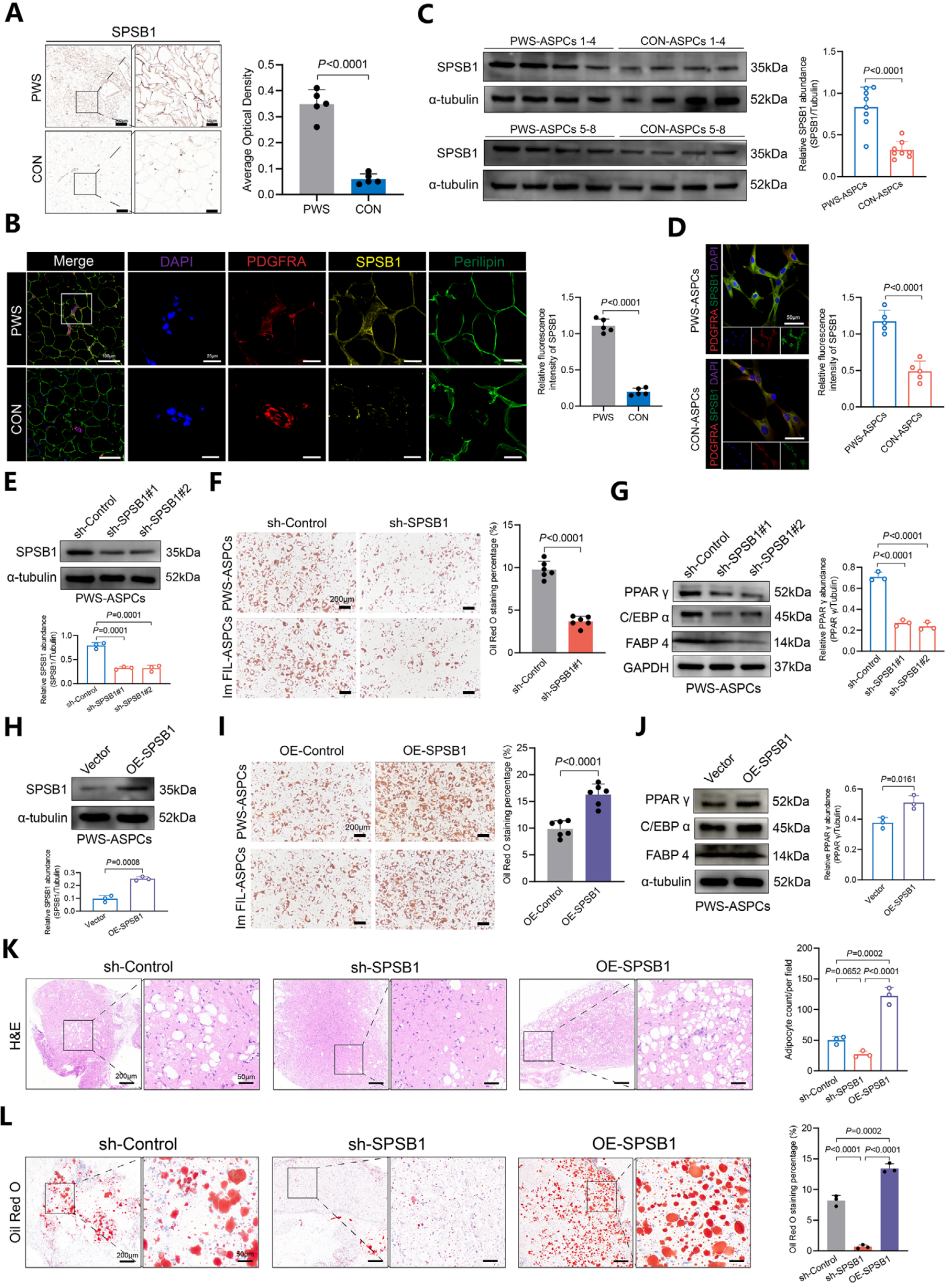
SPSB1 promoted adipogenesis in vitro and in vivo. (A): Higher levels of SPSB1 in PWS adipose tissues in comparison with CON adipose tissues by immunohistochemistry assay. (B): Representative immunofluorescence staining of PWS and CON adipose tissue. (C): WB and quantitative analysis of SPSB1 expression in PWS‐ASPCs and CON‐ASPCs. (D): Immunofluorescence analysis of SPSB1 protein level. (E): WB showing SPSB1 expression in ASPCs upon SPSB1 knockdown. (F): Oil Red O staining was used to evaluate the effect of SPSB1 knockdown on lipid droplet accumulation at day 8 of adipogenesis. Scale bar: 200 µm. (G): WB analysis showed the protein levels of PPAR γ, C/EBP α, and FABP 4 upon SPSB1 knockdown compared with the wild type at day 3 of adipogenesis. (H): WB showing SPSB1 expression in ASPCs upon SPSB1 overexpression. (I): Oil Red O staining was used to evaluate the effect of SPSB1 overexpression on lipid droplet accumulation at day 8 of adipogenesis. Scale bar: 200 µm. (J): WB analysis showed the protein levels of PPAR γ, C/EBP α, and FABP 4 upon SPSB1 overexpression compared with the wild type at day 3 of adipogenesis. (K): H&E staining of Matrigel implants collected on day 28 and quantification of adipocytes size. Scale bar: left: 200 µm, right: 50 µm. (L): Oil Red O staining of Matrigel implants from three groups and quantification of Oil red O staining area. Scale bar: left: 200 µm, right: 50 µm. Data were analyzed by unpaired two‐sided Student's t tests (A, B, C, D, F, H, I, J) or one‐way ANOVA (E, G, K, L). WB analysis were presented as mean ± SD with three replicate experiments. Original blot can be found in Figure S10.

Given that SPSB1^+^ ASPCs represented a distinct subpopulation unique to hypertrophic subcutaneous fat in PWS and exhibited high differentiation potential, we sought to further explore the impact of SPSB1 on the disease phenotype. Immunohistochemistry showed significantly higher expression of SPSB1 in PWS adipose tissue (Figure 3A). Immunofluorescence staining further confirmed elevated SPSB1 levels in both adipocytes and ASPCs within PWS adipose tissue (Figure 3B). Subsequently, we isolated ASPCs from PWS and control adipose tissues, and detected increased SPSB1 protein levels in PWS‑ASPCs (Figure 3C,D). We next investigated the role of SPSB1 in adipogenesis. Knockdown of SPSB1 impaired adipogenesis in both PWS‑ASPCs and Im‑FIL‑ASPCs, as evidenced by reduced lipid synthesis and lower expression of adipogenic markers (Figure 3E–G, Figure S3A,B) [31]. Conversely, overexpression of SPSB1 promoted adipogenesis in these cells (Figure 3H–J, Figure S3C,D).

To evaluate the in vivo role of SPSB1 in adipogenesis, a lipoma model was initially generated using PWS‑ASPCs [32]. Four weeks post‑implantation, the grafts were collected and processed for histological examination. H&E staining indicated that knockdown of SPSB1 led to a decrease in the population of mature adipocytes within the grafts, whereas enforced SPSB1 expression elevated adipocyte numbers (Figure 3K). Oil Red O staining further showed that SPSB1 silencing reduced lipid accumulation within the grafts, while SPSB1 overexpression enhanced lipid deposition (Figure 3L). To directly interrogate whether SPSB1 influenced adipogenesis in vivo, we placed Spsb1 under transcriptional control of the FABP4 promoter and packaged it into AAV8 particles. These viral vectors, either AAV8‑FABP4‑SPSB1‑eGFP or the control AAV8‑FABP4‑Scramble‑eGFP, were delivered systemically via tail vein injection. After six weeks, the animals were euthanized, and adipose depots were harvested alongside other relevant measurements (Figure S3E). Successful and specific transduction of adipocytes was evidenced by the co‑localization of eGFP fluorescence with adipocyte markers, confirming effective AAV‑mediated gene delivery into these cells (Figure S3F). Relative to control animals, mice receiving the SPSB1‑encoding virus displayed a striking increase in SPSB1 fluorescence intensity specifically within adipocytes (Figure S3G). Moreover, immunoblotting assays confirmed that SPSB1 protein abundance was substantially higher in adipocytes isolated from AAV8‑SPSB1‑treated mice (Figure S3H). These collective observations established that AAV8 could efficiently drive Spsb1 overexpression in adipocytes, culminating in elevated SPSB1 protein levels. Although animals in the SPSB1 group trended toward a modest increase in total body weight compared with controls, this difference failed to achieve statistical significance (Figure S3I, J). Additionally, no appreciable differences emerged in a panel of serum biochemical parameters between the two experimental groups (Figure S3K). Nonetheless, both subcutaneous and visceral fat depot masses were significantly higher in the SPSB1‑overexpressing cohort (Figure S3L, M). H&E staining further indicated that adipocytes residing in both fat depots exhibited enlarged cell sizes following SPSB1 overexpression relative to control mice (Figure S3N, O). Immunofluorescence analysis additionally showed that SPSB1 upregulation enhanced the expression of PPARγ within adipose tissues (Figure S3P). Taken together, these data supported a role for SPSB1 as a positive regulator of adipogenesis and lipid storage under both in vitro and in vivo conditions.

### 2.4 HDAC1 Was Identified as a New Substrate of SPSB1

**FIGURE 4:**
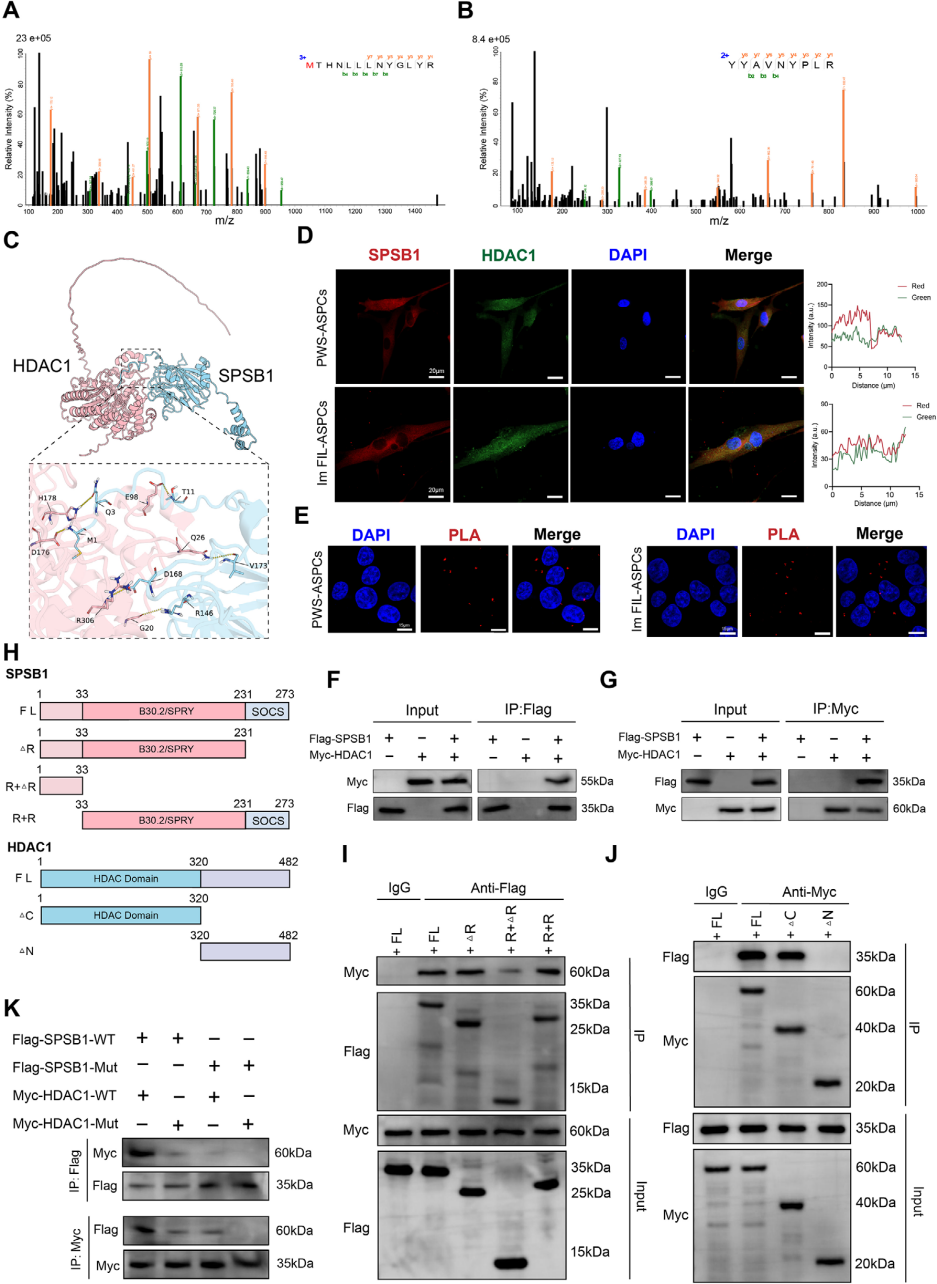
SPSB1 physically interacted with HDAC1. (A, B): Two peptides of HDAC1 identified by Immunoprecipitation‐mass spectrometry (IP‐MS) analysis. Enriched peptide sequence: (A) MTHNLLLNYGLYR (37‐49), (B) YYAVNYPLR (221‐229). (C): The predicted model of the protein complex was obtained through computation via the AlphaFold3 server. The output file was further analyzed with PyMOL, and detailed images of the binding interface between the two proteins were generated. (D): SPSB1 and HDAC1 were detected by immunofluorescence staining in PWS‐ASPCs and Im FIL‐ASPCs (left). The co‐localization analysis was performed by Image J (right). The red line indicated the region used for measuring the colocalization signal. (E): Proximity ligation assays (PLA) were performed using anti‐SPSB1 and anti‐HDAC1 antibodies in PWS‐ASPCs and Im FIL‐ASPCs. Red signals represent PLA signals, indicating the proximity of the targeted proteins, while blue signals denote the cell nuclei. (F, G): HEK293T cellular lysates were analyzed by Co‐IP followed by western blotting. (H): Schematic representation of SPSB1 and HDAC1 truncations. (I): IP and western blot assay of the interaction between FLAG‐tagged truncated SPSB1 and Myc‐tagged HDAC1 in HEK293T cells. Cell extracts were IP with an anti‐Flag antibody. (J): IP and western blot assay of the interaction between FLAG‐tagged SPSB1 and Myc‐tagged truncated HDAC1 in HEK293T cells. Cell extracts were IP with an anti‐Myc antibody. (K): IP and western blot assay showed the interactions between FLAG‐tagged mutated SPSB1 and Myc‐tagged mutated HDAC1 in HEK293T cells. Original blot can be found in Figure S10.

Given the core function of SPSB1 in “tagging” specific substrate proteins for the ubiquitin‐proteasome system, thereby regulating their stability and activity, we aimed to identify its specific substrates. Immunoprecipitation – mass spectrometry (IP‑MS) analysis performed on PWS‐ASPCs overexpressing SPSB1 suggested that HDAC1 may be an interacting partner of SPSB1, as the IP‐MS results enriched multiple distinct peptides of HDAC1 (Figure 4A,B). This candidate was selected based on a prior report showing that POU2AF1 promoted adipogenesis of mesenchymal stem cells by inhibiting HDAC1 expression [33]. We first asked whether SPSB1 physically associated with HDAC1. In‐silico docking predicted a compatible interface involving multiple amino‐acid contacts distributed across both proteins (Figure 4C, Figure S5A). To evaluate the thermodynamic and kinetic stability of the SPSB1‐HDAC1 complex, we conducted 10‐ns all‐atom molecular‐dynamics (MD) simulations in explicit solvent. In the RMSD–time curve, the system continued to rise slowly from 1 ns to 3 ns, indicating a progressive equilibration process (Figure S4A). RMSF analysis showed that the core region of HDAC1 (residues ≈20–430) and the main body of SPSB1 (residues 20‐235) remained stable, with fluctuations between 0.5 and 1.0 nm (Figure S4B, C). The radius of gyration (Rg) curve revealed a slight expansion of the system during the first 3–4 ns, followed by a plateau from 4–7 ns and subsequent convergence, reflecting a conformational tightening from a loose to a more compact state (Figure S4D). Changes in solvent‐accessible surface area (SASA) were consistent with this trend: initial fluctuations were followed by a decrease at 6–8 ns and stabilization thereafter, indicating gradual burial of hydrophobic interfaces and tighter binding (Figure S4E, F). The minimal interfacial distance remained around 0.16 nm throughout the simulation, confirming that the two proteins maintained close contact without dissociation or non‐physical clashes (Figure S4G). The free energy landscapes (2D and 3D Gibbs Energy Landscape) further revealed that the conformational distribution of the system was concentrated in a curved low‐energy valley at PC1 ≈ 1.0‐1.4 and PC2 ≈ 3.3‐3.7, with multiple blue‐green local minima corresponding to metastable basins, suggesting transitions among several closely related stable conformations (Figure S4H, I). In summary, the core regions of HDAC1 and SPSB1 remained stable, with a robust interfacial contact and a stable hydrogen‐bond network. The RMSD, Rg, SASA, and free energy analyses consistently indicated that the protein‐protein complex formed a stable binding interface and an energetically favorable conformation during the later stages of simulation, providing a plausible molecular framework for subsequent functional interrogation.

Using immunofluorescence staining, we observed overlapping subcellular localization signals between SPSB1 and HDAC1 (Figure 4D). A proximity ligation assay (PLA) subsequently captured a transient, endogenous interaction between these two proteins (Figure 4E). In heterologous overexpression settings using 293T cells, Co‑IP experiments confirmed that exogenously supplied flag‑tagged SPSB1 and myc‑tagged HDAC1 could physically interact with each other (Figure 4F,G). For domain mapping, we dissected full‑length SPSB1 into three truncated fragments and full‑length HDAC1 into two segments, based on their respective domain architectures (Figure 4H). Through Co‑IP mapping, we identified the SPRY domain of SPSB1 (spanning residues 33–231) as necessary and sufficient for binding to HDAC1, whereas the interacting region on HDAC1 was localized to its N‑terminal half (residues 1–320) (Figure 4I,J). Reciprocal Co‑IP assays independently validated these observations, demonstrating that HDAC1 (1–320) could reciprocally precipitate SPSB1 (33–231) (Figure S5B, C). Computational docking simulations had previously suggested potential contacts between residues 82 and 168 of SPSB1 and residues 176 and 306 of HDAC1 (Figure 4C). Mutagenesis experiments revealed that alanine substitutions at these positions—whether introduced individually into the predicted binding interface of SPSB1 or HDAC1, or introduced simultaneously into both proteins—effectively abrogated their physical interaction (Figure 4K). Collectively, these results identified HDAC1 as a previously unrecognized interactor and substrate of SPSB1.

### 2.5 SPSB1‐Mediated K29 and K48‐linked Ubiquitination Promoted HDAC1 Degradation

**FIGURE 5:**
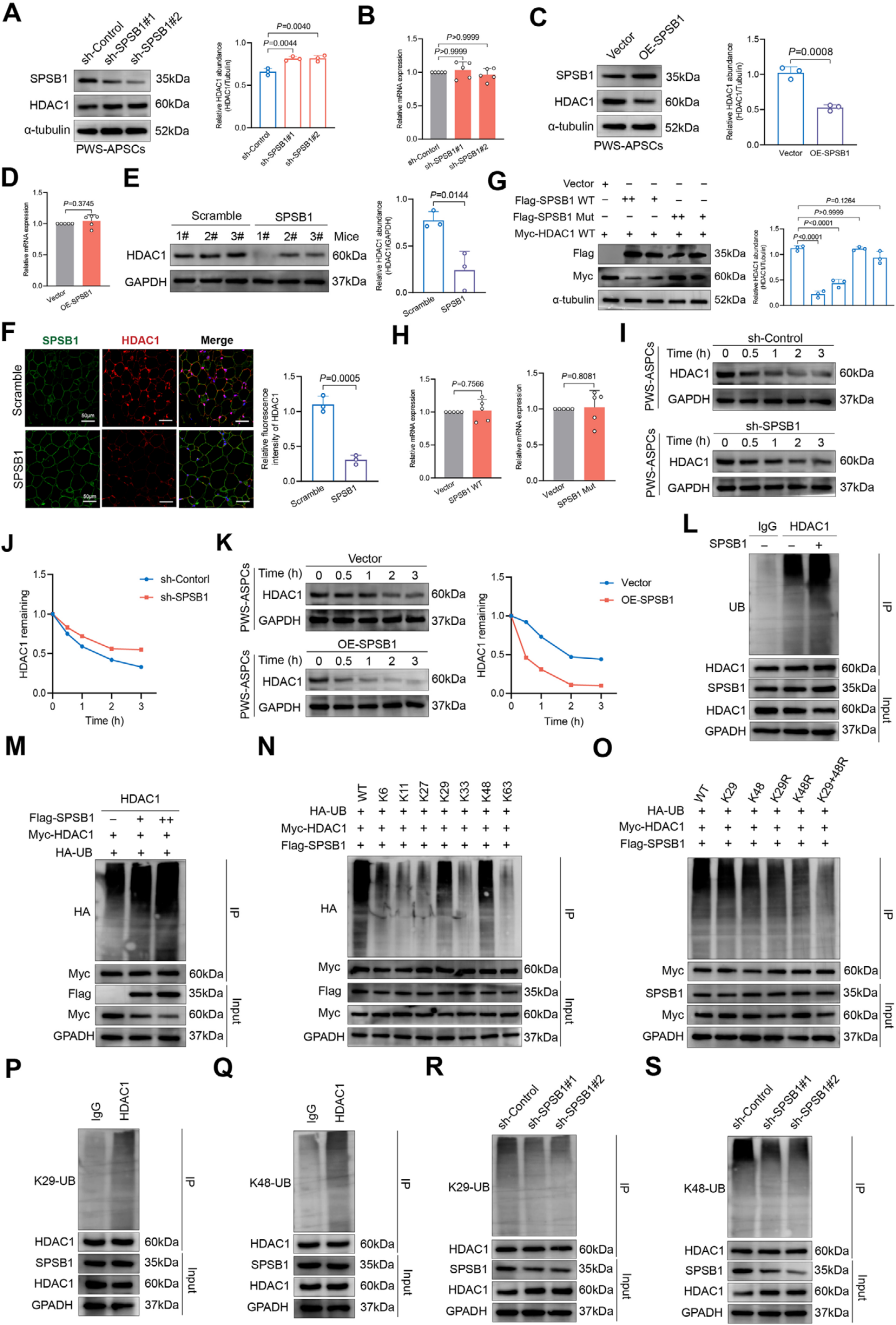
SPSB1 mediated the K29‐ and K48‐linked polyubiquitination of HDAC1. (A): WB and quantitative analysis showed the protein levels of HDAC1 upon SPSB1 knockdown in PWS‐ASPCs. (B): qPCR analysis showed that the mRNA levels of HDAC1 upon SPSB1 knockdown in PWS‐ASPCs. (C): WB and quantitative analysis showed the protein levels of HDAC1 upon SPSB1 overexpression in PWS‐ASPCs. (D): qPCR analysis showed that the mRNA levels of HDAC1 upon SPSB1 overexpression in PWS‐ASPCs. (E): WB and quantitative analysis of HDAC1 protein expression in the adipose tissue obtained from wild‐type and adipose specific SPSB1‐overexpression mice. (F): Immunofluorescence staining for HDAC1 in the adipose tissue obtained from wild‐type and adipose specific SPSB1‐overexpression mice. (G): HEK293T cells were transfected with plasmids encoding Myc‐tagged HDAC1, along with a plasmid encoding Flag‐tagged wild‐type SPSB1 or SPSB1 mutants. Cell lysates were analyzed by WB with indicated antibodies. (H): HEK293T cells were transfected with a plasmid encoding Flag‐tagged wild‐type SPSB1 or SPSB1 mutants. Cell lysates were analyzed by qPCR. (I–K): PWS‐ASPCs were transfected with the indicated lentivirus. After transfection, cells were treated with 100 µg/mL cycloheximide (CHX) and collected for immunoblot analysis at the indicated time points. (L): PWS‐ASPCs with or without SPSB1 overexpression were treated with MG132 for 4 h. Cell lysates were analyzed by IP with anti‐HDAC1 antibody and WB with indicated antibodies. (M): HEK293T cells were transfected with the indicated plasmids. After transfection, cells were treated with MG132 for 4 h. Cell lysates were analyzed by IP with anti‐Myc antibody and WB with indicated antibodies. (N, O): Co‐immunoprecipitation analysis of HDAC1 polyubiquitination in HEK293 cells transfected with expression plasmids for Flag‐SPSB1 and HA‐ubiquitin‐WT or its mutants (only a single lysine residue was retained). (P, Q): WB analysis of whole cellular lysates and IPs derived from lysates of PWS‐ASPCs using indicated K29‐UB or K48‐UB antibodies. Cells were treated with 20 µM MG132 for 4 h before harvesting. (R, S): WB analysis of whole cellular lysates and anti‐HDAC1 IPs derived from PWS‐ASPCs stably expressing sh‐SPSB1. Cells were treated with 20 µM MG132 for 4 h before harvesting. Data were analyzed by unpaired two‐sided Student's t tests (C, D, E, F, H) or one‐way ANOVA (A, B, G). and were presented as mean ± SD with at least three replicate experiments. Original blot can be found in Figure S10.

Given that SPSB1 functions as an adapter subunit of the ECS complex to facilitate ubiquitination and is widely involved in the degradation of functional proteins such as HnRNPA1 and TβRII, we hypothesized that HDAC1 could be a novel target of SPSB1. To further demonstrate the role of SPSB1 in regulating HDAC1 expression, we knocked down SPSB1 in PWS‑ASPCs and Im‑FIL‑ASPCs using two independent shRNAs. The results showed that SPSB1 knockdown led to a significant increase in HDAC1 protein abundance without altering its mRNA levels (Figure 5A,B and Figure S6A,B). Conversely, overexpression of SPSB1 reduced HDAC1 protein levels without affecting mRNA expression (Figure 5C,D and Figure S6C,D). Consistent with these findings, HDAC1 levels were markedly decreased in adipose tissue of AAV8‑SPSB1‑SLC7A11‑eGFP mice (Figure 5E,F). Overexpression of wild‑type SPSB1 reduced HDAC1 levels, whereas a mutant form of SPSB1 unable to interact with HDAC1 lost this effect, with no significant changes observed in HDAC1 mRNA (Figures 4K,  5G,H and Figure S6E,F). Moreover, in the presence of cycloheximide (CHX), SPSB1 accelerated HDAC1 degradation, while SPSB1 knockdown slowed it down (Figure 5I–K and Figure S6G–I). Correspondingly, SPSB1 overexpression increased the ubiquitination level of HDAC1 (Figure 5L,M).

To determine the types of ubiquitin linkages assembled on HDAC1, we co‐transfected HDAC1 with seven lysine (K)‐only ubiquitin constructs, each retaining only the specified lysine residue while the other six lysines were mutated to arginine (R). These lysine‐only ubiquitin mutants allowed us to assess which inter‐ubiquitin linkage types are preferentially utilized in SPSB1‐mediated HDAC1 ubiquitination. The experiments demonstrated that SPSB1 overexpression predominantly promoted K29‐linked and K48‐linked polyubiquitin chain assembly on HDAC1 (Figure 5N). Introduction of ubiquitin constructs harboring K29R or K48R mutations reduced HDAC1 ubiquitination, and this effect was even more pronounced when both K29R and K48R mutations were combined (Figure 5O). Moreover, endogenous HDAC1 was found to be ubiquitinated by K29‐linked and K48‐linked polyubiquitin chains in both PWS‑ASPCs and Im‑FIL‑ASPCs (Figure 5P, Q and Figure S6J, K). Knockdown of SPSB1 significantly decreased endogenous K29‐linked and K48‐linked ubiquitination of HDAC1, indicating that SPSB1 specifically regulated this modification (Figure 5R, S and Figure S6L, M). To examine whether components of the ECS complex were involved in SPSB1‐mediated HDAC1 ubiquitination, we depleted them in 293T cells by transfection with specific siRNAs. Knockdown of CUL5 reduced the ubiquitination level of HDAC1, and concurrent knockdown of Elongin B and CUL5 further exacerbated this effect (Figure S6N). In summary, these findings indicated that SPSB1 bind to HDAC1 and recruited the ECS complex to enhance K29‐linked and K48‐linked polyubiquitin chain formation on HDAC1, thereby promoting its degradation.

### 2.6 Ubiquitination of HDAC1 at K90 and K361 Depended on the SOCS Domain of SPSB1

**FIGURE 6:**
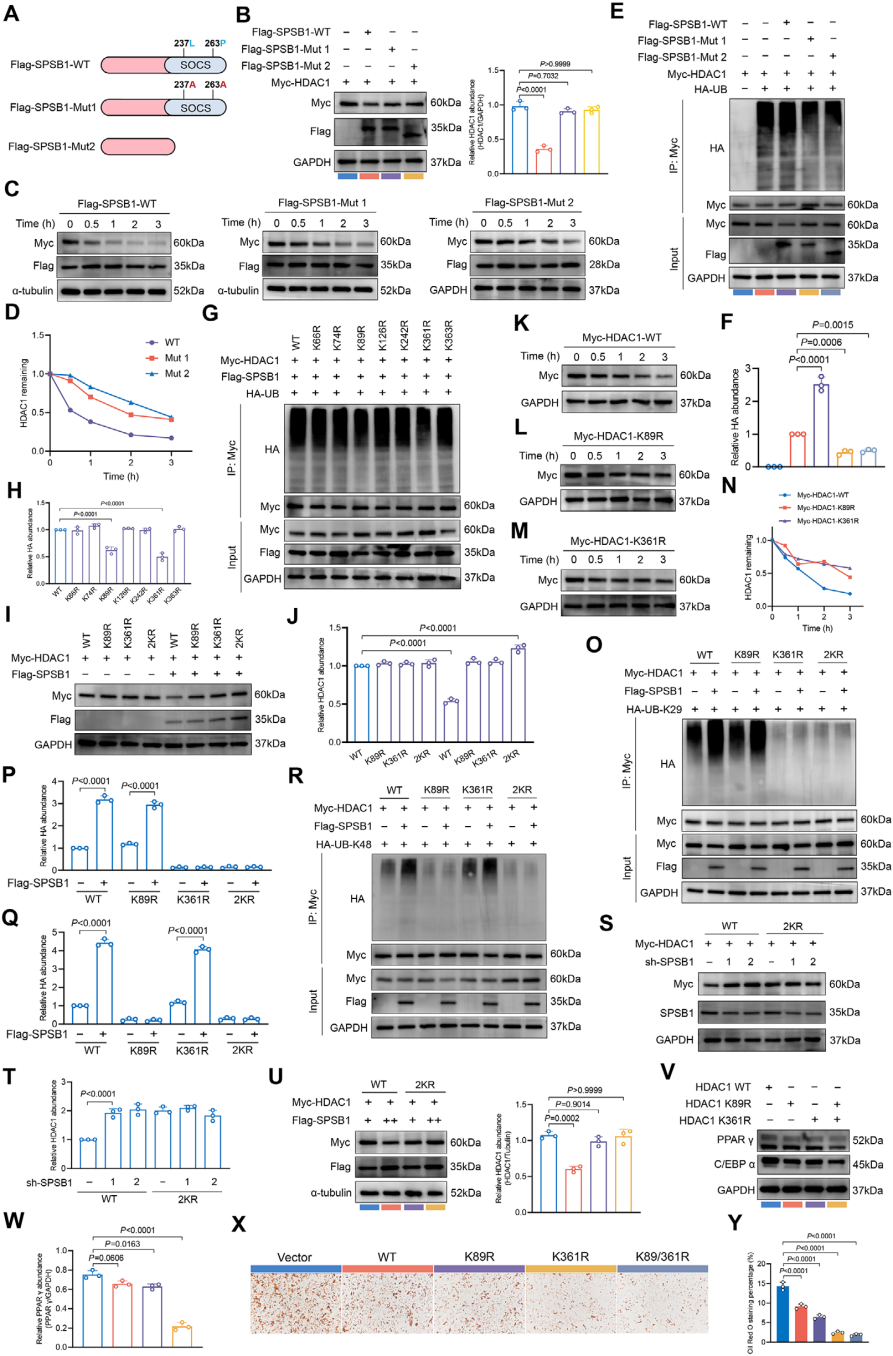
Ubiquitination of HDAC1 at K89 and K361 depended on the SOCS domain of SPSB1. (A): The schematic diagram of wild‐type and mutant SPSB1. (B): HEK293T cells were transfected with plasmids encoding Myc‐tagged HDAC1, along with a plasmid encoding Flag‐tagged wild‐type SPSB1 or SPSB1 mutants (M1, M2). Cell lysates were analyzed by western blot with indicated antibodies. (C, D): HEK293T cells were transfected with the indicated plasmids. After transfection, cells were treated with 100 µg/mL CHX and collected for immunoblot and quantitative analysis at the indicated time points. (E, F): HEK293T cells were transfected with the indicated plasmids. After transfection, cells were treated with MG132 for 4 h. Cell lysates were analyzed by IP with anti‐Myc antibody and WB with indicated antibodies. (G, H): Wild‐type and lysine residual mutated Myc‐tagged HDAC1 plasmids were individually transfected into HEK293T cells with Flag‐tagged SPSB1. Cell lysates were analyzed by western blot with indicated antibodies. (I, J): WB and quantitative (J) analysis of HEK293T cells transfected with Myc‐HDAC1 (WT) or mutations (K89R, K361R, or K89 /361R), and Flag‐SPSB1. (K–N): HEK293T cells were transfected with Myc‐HDAC1‐WT, Myc‐HDAC2‐K89R or Myc‐HDAC2‐K361R plasmid and then treated with CHX for the indicated times before harvesting. Cell lysates were analyzed by immunoblotting with indicated antibodies and quantification (N) of HDAC1 band intensity. (O–R): Coimmunoprecipitation and quantitative (P, Q) analysis of polyubiquitination in HEK293T cells transfected with HA‐tagged K29‐(O) or K48‐(R) UB and the indicated Myc‐HDAC1 mutants in presence of Flag‐SPSB1 or its empty vector as control. (S, T): Immunoblot and quantitative (T) analysis of Myc‐HDAC1 or Myc‐HDAC1‐2KR in their stable expressing HEK293T cells transfected with a shRNA targeting SPSB1. (U): Immunoblot and quantitative analysis of Myc‐HDAC1 or Myc‐HDAC1‐2KR in their stable expressing HEK293T cells with SPSB1 overexpression. (V, W): WB and quantitative (W) analysis showed that the protein levels of PPAR γ and C/EBP α upon HDAC1 mutants transfection at day 3 of adipogenesis. (X, Y): Oil Red O staining was used to evaluate the effect of HDAC1 mutants on lipid droplet accumulation at day 8 of adipogenesis. Scale bar: 200 µm. Data were analyzed by one‐way ANOVA (B, F, H, J, P, Q, T, U, W, Y) and were presented as mean ± SD with at least three replicate experiments. Original blot can be found in Figure S10.

As a component of the ECS complex, SPSB1 interacts with Elongin B/C and Cul2/5 via its SOCS domain (residues 232–273), with leucine 237 and proline 263 identified as critical residues for binding [16]. Accordingly, we generated two mutants: mutant 1 (M1), in which these two residues were substituted with alanine; and mutant 2 (M2), which lacks the entire SOCS domain (Figure 6A). Overexpression of wild‑type SPSB1 decreased HDAC1 protein levels, whereas neither M1 nor M2 produced this effect (Figure 6B). Moreover, transfection of M1 or M2 significantly slowed HDAC1 degradation (Figure 6C,D). Subsequent experiments showed that while SPSB1 overexpression increased HDAC1 ubiquitination, but introduction of M1 or M2 markedly reduced this modification (Figure 6E,F). Together, these results further confirmed that SPSB1 accelerated HDAC1 degradation through its SOCS domain.

Next, we aimed to identify the specific lysine residues on HDAC1 itself that undergo ubiquitination. We queried the Protein Lysine Modification Database (http://plmd.biocuckoo.org/) and performed single‑point mutations targeting the most probable ubiquitination sites on HDAC1. Western blot analysis revealed that mutating either K89 or K361 to arginine impaired SPSB1‑mediated ubiquitination of HDAC1 (Figure 6G,H). Similarly, mutation of HDAC1 K89 or K361 to arginine prevented SPSB1‑induced HDAC1 degradation (Figure 6I,J). Introduction of either K89R or K361R mutation in HDAC1 also extended the half‑life of HDAC1 (Figure 6K–N). To determine which ubiquitin linkage type was conjugated to each specific HDAC1 lysine residue, we performed linkage‐specific ubiquitination assays. These experiments demonstrated that K48‐linked polyubiquitin chains were conjugated to K89 of HDAC1, whereas K29‐linked polyubiquitin chains were conjugated to K361 of HDAC1 (Figure 6O–R). Additionally, we generated HEK293 cells stably expressing either HDAC1‑WT (wild‑type) or HDAC1‑2KR (a double mutant with K89 and K361 both mutated to arginine). Overexpression of SPSB1 promoted degradation of HDAC1‑WT, while SPSB1 knockdown increased its expression. However, SPSB1 had no effect on the protein level of HDAC1‑2KR (Figure 6S–U). HDAC1 has been clearly reported to inhibit adipogenic differentiation. We observed that overexpression of HDAC1 reduced lipid accumulation, and this inhibitory effect was more pronounced with HDAC1 mutants carrying K89R and/or K361R substitutions (Figure 6X,Y). Western blotting showed that overexpression of K89R or K361R mutated HDAC1 suppressed expression of adipogenic markers more strongly than wild‑type HDAC1 (Figure 6V–W), likely due to the higher intracellular protein levels of the mutant forms. Taken together, these data indicated that SPSB1 promoted K29‐linked polyubiquitination at K361 of HDAC1 and K48‐linked polyubiquitination at K89 of HDAC1, respectively.

### 2.7 SPSB1 Promoted the Degradation of HDAC1 in Dual Proteolytic Pathways

**FIGURE 7:**
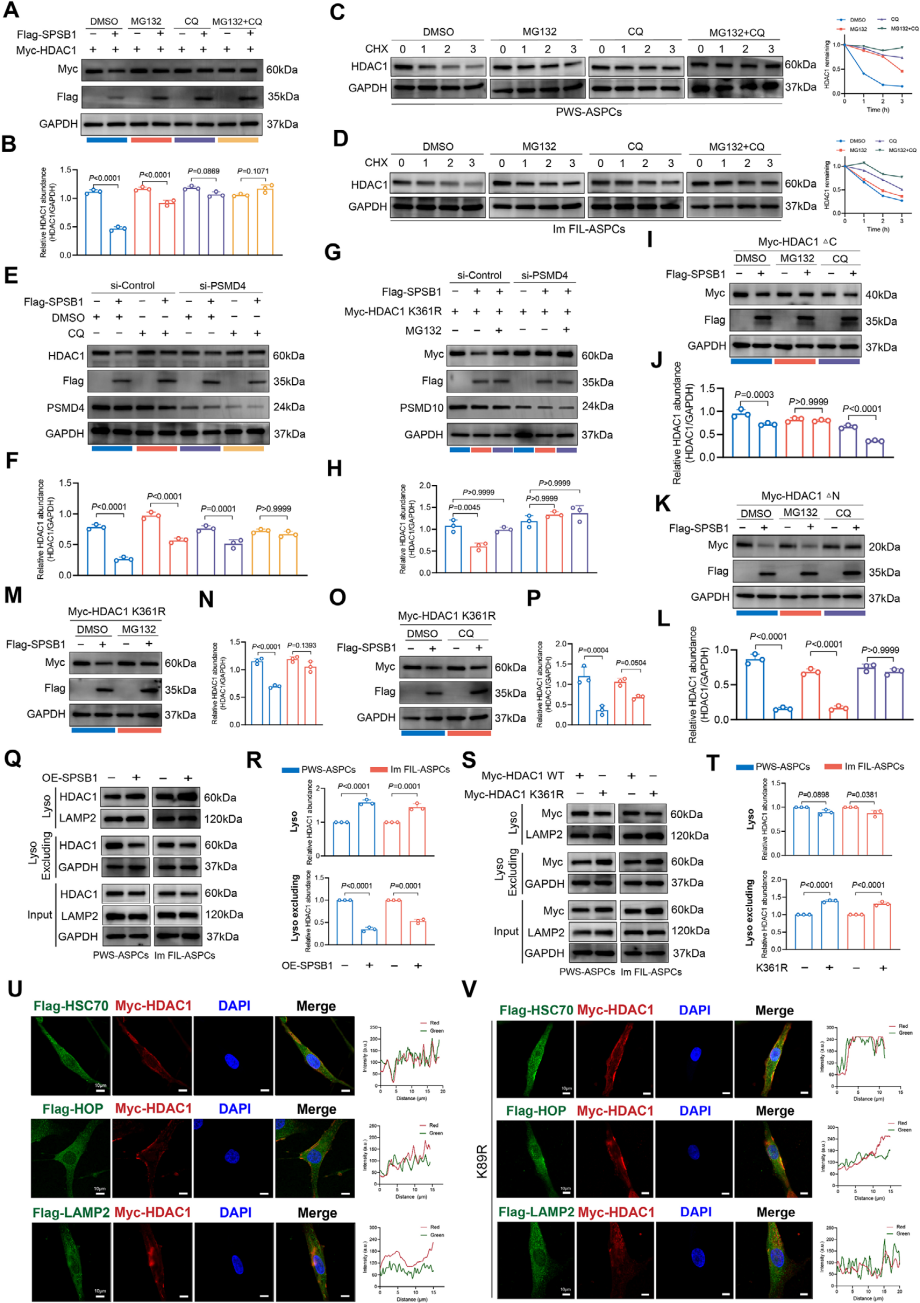
SPSB1 promoted the degradation of HDAC1 in two proteolytic pathways. (A, B): WB and quantitative (B) analysis of HEK293 cells transfected with expression plasmids of Myc‐HDAC1 and Flag‐SPSB1 or its empty vector and then treated with DMSO, MG132, CQ, or MG132 plus CQ. (C, D): The degradation of HDAC1 in PWS‐ASPCs (C) or Im FIL‐ASPCs (D) treated with DMSO, MG132, CQ, or MG132 plus CQ was evaluated by CHX‐chase assay. Right, quantification of the intensity determined by the relative level of HDAC1remaining. (E, F): Immunoblot and quantitative (F) analysis of HDAC1 in PSMD4 WT and knockdown HEK293T cells transfected with Flag‐SPSB1 or empty vector and then treated with DMSO or CQ. (G, H): Immunoblot and quantitative (H) analysis of HDAC1 in PSMD4 WT and knockdown HEK293T cells transfected with Myc‐HDAC1 K361R and Flag‐SPSB1 or empty vector and then treated with DMSO or MG132. (I–L): Immunoblot and quantitative (J, L) analysis of HEK293 cells transfected with Myc‐HDAC1 △C or △N with Flag‐SPSB1 or empty vector and then treated with DMSO, MG132 or CQ. (M–P): Immunoblot and quantitative (N, P) analysis of HEK293 cells transfected with Myc‐HDAC1 K361R with Flag‐SPSB1 or empty vector and then treated with DMSO, MG132 or CQ. (Q, R): Cellular fractions were subjected to western blot and quantitative analysis (R) to compare HDAC1 abundance in lysosomal and extra‐lysosomal compartments in PWS‐ASPCs and Im FIL‐ASPCs with or without SPSB1 overexpression. (S, T): Cellular fractions were subjected to western blot and quantitative analysis (T) to compare HDAC1 abundance in lysosomal and extra‐lysosomal compartments in PWS‐ASPCs and Im FIL‐ASPCs with or without Myc‐HDAC1 K361R transfection. (U): Myc‐HDAC1 and Flag‐HSC70, Flag‐HOP, or Flag‐LAMP2 were detected by immunofluorescence staining in Im FIL‐ASPCs (left). The co‐localization analysis was performed by Image J (right). (V): Myc‐HDAC1 and Flag‐HSC70, Flag‐HOP, or Flag‐LAMP2 were detected by immunofluorescence staining in Im FIL‐ASPCs with Myc‐HDAC1 K89R mutant (left). The co‐localization analysis was performed by Image J (right). Data were analyzed by unpaired two‐sided Student's t tests (B, F, J, L, N, P, R, T) or one‐way ANOVA (H), and were presented as mean ± SD with at least three replicate experiments. Original blot can be found in Figure S10.

We next sought to determine the pathway through which SPSB1‐mediated polyubiquitination of HDAC1 regulated its degradation. Using the proteasome inhibitor MG132 and the lysosome inhibitor chloroquine (CQ) to block the UPS and ALP, respectively, we found that SPSB1 overexpression accelerated HDAC1 degradation. This degradation was partially delayed by either MG132 or CQ alone, but effectively blocked by their combined use, which restored HDAC1 levels (Figure 7A,B). Under basal conditions, both MG132 and CQ also delayed HDAC1 degradation, with a more pronounced effect upon co‑treatment (Figure 7C,D). PSMD4, a key subunit of the 26S proteasome, was then knocked down in Im FIL‐ASPCs. We observed that CQ alone partially blocked the SPSB1‑induced decrease in HDAC1, but CQ fully restored HDAC1 protein levels in PSMD4‐knockdown cells (Figure 7E,F). These results suggested that SPSB1‑mediated HDAC1 degradation may involve both the UPS and ALP. Given that K48‑linked ubiquitination at HDAC1 K89 has been clearly shown to promote UPS‑mediated degradation, we investigated whether K29‑linked ubiquitination at K361 might target HDAC1 for ALP. In cells expressing the K361R mutant, MG132 effectively blocked SPSB1‑mediated HDAC1 degradation, and this degradation was abolished upon additional PSMD4 knockdown (Figure 7G,H). Furthermore, MG132 treatment prevented degradation of HDAC domain (1‐320), whereas CQ treatment restored expression of the HDAC1 (321–482) fragment (Figures 7I–L, 4H). In cells expressing the K361R mutant, MG132 largely restored HDAC1 expression, but CQ lost this effect (Figure 7M–P). Subcellular fractionation followed by western blotting revealed that SPSB1 overexpression increased HDAC1 accumulation in lysosomes (Figure 7Q,R), whereas the K361R mutation reduced lysosomal HDAC1 levels (Figure 7S,T). These data indicated that K29‑linked ubiquitination at K361 promoted HDAC1 degradation via the lysosomal pathway.

To identify the adaptor involved in HDAC1 targeting to the ALP, we analyzed the HDAC1 sequence and identified potential KFERQ‑like motifs recognized by the chaperone HSC70 (Figure S7A). Notably, three overlapping motifs were located within residues 359–364, adjacent to the K361 ubiquitination site. HSC70 recognizes soluble cytosolic proteins bearing KFERQ‑like motifs and facilitates their delivery to lysosomes for degradation [34]. HOP facilitated substrate transfer from HSC70 to LAMP2, enabling lysosomal entry and subsequent degradation [35]. Co‑IP confirmed an interaction between HSC70 and HDAC1 (Figure S7B, C), and immunofluorescence showed co‑localization of HSC70, HOP, LAMP2, and HDAC1 (Figure 7U). The interaction between HSC70 and the HDAC1 K361R mutant was reduced compared with wild‑type HDAC1 (Figure S7D, E). Immunofluorescence further demonstrated that the K361R mutation, but not the K89R mutation, decreased co‑localization of HDAC1 with HSC70, HOP, and LAMP2 (Figure 7V, Figure S7F). Notably, CQ administration increased HDAC1 levels, but this effect was partially blocked after HSC70 knockdown (Figure S7G–J). This suggested that SPSB1‑mediated ubiquitination at K361 enhanced HSC70 recognition of HDAC1, thereby promoting its lysosomal degradation via chaperone‑mediated autophagy (Figure S7K). In summary, these results demonstrated that SPSB1 promoted polyubiquitination at K89 (K48‑linked) and K361 (K29‑linked) of HDAC1, subsequently directing the substrate to distinct degradation routes: the UPS and the ALP, respectively.

### 2.8 HDAC1 Suppressed Adipogenesis by Orchestrating Chromatin Accessibility and Epigenetic Remodeling

**FIGURE 8:**
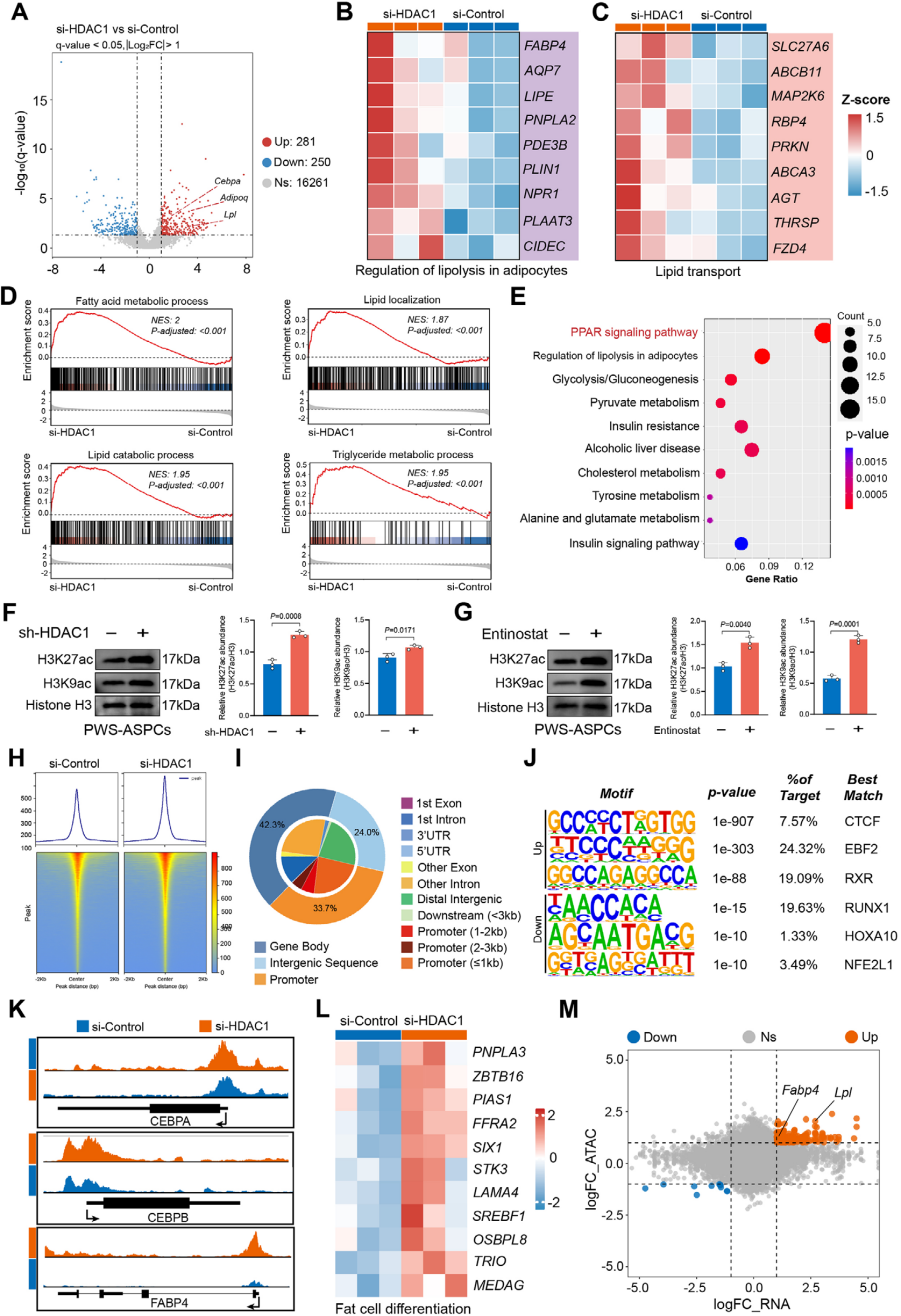
HDAC1 regulated adipogenesis by transcriptional reprogramming. (A): Volcano plot of significantly upregulated (red) and downregulated (blue) genes identified by comparing si‐HDAC1 or si‐Control treated PWS‐ASPCs. (B): Heatmaps showed the RNA‐seq results of differentially expressed genes related to regulation of lipid metabolic process in si‐HDAC1 or si‐Control treated PWS‐ASPCs. (C): Heatmaps showed the RNA‐seq results of differentially expressed genes related to lipid transport in si‐HDAC1 or si‐Control treated PWS‐ASPCs. (D): Gene set enrichment analysis plot of genes related to adipogenesis that were enriched in PWS‐ASPCs with HDAC1 knockdown. (E): Dot plot showed the significant Kyoto Encyclopedia of Genes and Genomes pathways among the differentially expressed genes. (F): WB and quantitative analysis for the expression of H3K27ac and H3K9ac in PWS‐ASPCs with or without HDAC1 knockdown. (G): WB analysis for the expression of H3K27ac and H3K9ac in PWS‐ASPCs treated with entinostat. (H): Heatmaps for differentially enriched ATAC‐seq peaks in PWS‐ASPCs with or without HDAC1 knockdown. (I): Pie chart showed the genomic distribution of accessible regions. (J): Motif enrichment analysis of differentially accessible chromatin regions in HDAC1‐knockdown PWS‐ASPCs. This analysis reported absolute motif frequencies within differential peaks and did not provide fold enrichment relative to controls due to methodological limitations of standard motif discovery algorithms. (K): IGV maps showed the increased ATAC‐seq peaks of representative adipogenic genes. (L): Heatmaps showed the ATAC‐seq analysis of upregulated genes related to fat cell differentiation. (M): Quadrantal diagram depicting the overlap of significantly differentially expressed genes from bulk RNA‐seq and genes with significantly variational peaks from ATAC‐seq data. Both upregulated (orange); both downregulated (blue). Data were analyzed by unpaired two‐sided Student's t tests (F, G) and were presented as mean ± SD with at least three replicate experiments. Original blot can be found in Figure S10.

To dissect the molecular events through which HDAC1 exerted control over adipogenesis, we conducted RNA‑sequencing on PWS‑ASPCs subjected to adipogenic induction following HDAC1 knockdown (GSA‐Human: HRA016261). Knockdown of HDAC1 triggered a widespread alteration in the transcriptome, characterized by 281 genes showing increased expression and 250 genes showing decreased expression, implying that HDAC1 acted as a global transcriptional regulator during adipogenesis (Figure 8A). Notably, transcripts encoding key adipogenic determinants, including cebpa, were found to be upregulated (Figure 8A). In keeping with the phenotypic outcomes, genes whose expression rose after HDAC1 depletion—such as Fabp4, Slc27a6, and Lipe—had established functions in adipocyte lipolysis regulation and lipid transport (Figure 8B,C). Gene set enrichment analysis (GSEA) demonstrated that the genes most significantly altered in HDAC1‑knockdown cells were preferentially enriched in pathways governing fatty acid metabolism (Figure 8D). KEGG pathway analysis further pinpointed that these differentially expressed transcripts converge on multiple signaling cascades previously implicated in adipocyte differentiation, prominently including the PPAR signaling pathway (Figure 8E).

HDAC1 enzymatically removes acetyl moieties from lysine side chains on histones, thereby promoting a compacted, transcriptionally silent chromatin configuration [36]. Among the various acetylation marks, H3K27ac and H3K9ac (serving as hallmarks of active transcription) are particularly sensitive to HDAC1 activity [37, 38]. Knockdown of HDAC1, as well as pharmacological inhibition using entinostat, led to elevated global levels of both H3K9ac and H3K27ac in PWS‑ASPCs and Im FIL‑ASPCs (Figure 8F,G, Figure S8A, B). By contrast, knockdown of SPSB1 diminished these two histone modifications, an effect attributable to the ability of SPSB1 to antagonize HDAC1 ubiquitination, thereby preserving HDAC1 protein stability (Figure S8C, D). ChIP‑qPCR assays subsequently confirmed that HDAC1 knockdown resulted in increased deposition of H3K27ac and H3K9ac at the promoter regions of adipogenic genes (Figure S8E, F). A negative correlation between HDAC1 mRNA levels and the expression of adipogenic genes in adipose tissue was further corroborated by mining the Chipbase database (Figure S8G).

To gain deeper insight into the epigenetic layer of HDAC1‑mediated regulation, we performed ATAC‑seq on PWS‑ASPCs after HDAC1 knockdown (GSA‐Human: HRA016265). Genome‑wide profiling of ATAC‑seq signal enrichment in regions surrounding transcription start sites (TSS ± 2 kb) revealed that HDAC1 depletion substantially increased overall chromatin accessibility (Figure 8H). When we annotated the differentially accessible peaks according to their genomic locations, the majority of gained peaks mapped to gene body and promoter regions (Figure 8I). Motif enrichment analysis of differentially accessible chromatin regions in HDAC1‐KD PWS‐ASPCs revealed an enrichment of pro‐adipogenic transcription factor binding motifs within regions of increased accessibility, and anti‐adipogenic transcription factor motifs within regions of decreased accessibility, consistent with a chromatin landscape favoring adipogenic commitment (Figure 8J) [39, 40, 41, 42, 43, 44]. Importantly, mirroring the RNA‑seq results, ATAC‑seq browser tracks illustrated that promoter regions of established adipogenic regulators (Cebpa, Cebpb, and Fabp4) as well as genes involved in fat cell differentiation (Zbtb16 and Pias1) displayed markedly heightened accessibility in HDAC1‑knockdown cells compared to controls (Figure 8K,L). Seeking to connect accessibility changes with transcriptional outcomes, we integrated the ATAC‑seq and RNA‑seq datasets. This integrative analysis uncovered 77 overlapping genes whose expression and promoter accessibility were concordantly altered, among which 68 were upregulated and 9 were downregulated (Figure 8M). Several of the concordantly upregulated genes, known to promote adipogenic differentiation, exhibited more open promoter configurations upon HDAC1 knockdown (Figure 8M). To interpret the functional implications of the observed chromatin accessibility shifts, we performed GO and KEGG enrichment analyses on genes linked to differentially accessible regions. GO terms associated with lipid metabolism and adipocyte differentiation were significantly upregulated, while KEGG analysis again highlighted a robust enrichment of the PPAR signaling pathway following HDAC1 knockdown (Figure S8H, I). Collectively, these findings supported a model wherein reduced HDAC1 levels in PWS‑ASPCs facilitated adipogenesis by increasing chromatin accessibility and transcriptional activation of adipogenic genes.

### 2.9 HDAC1 Mediated the Adipogenesis‐Promoting Role of SPSB1

**FIGURE 9:**
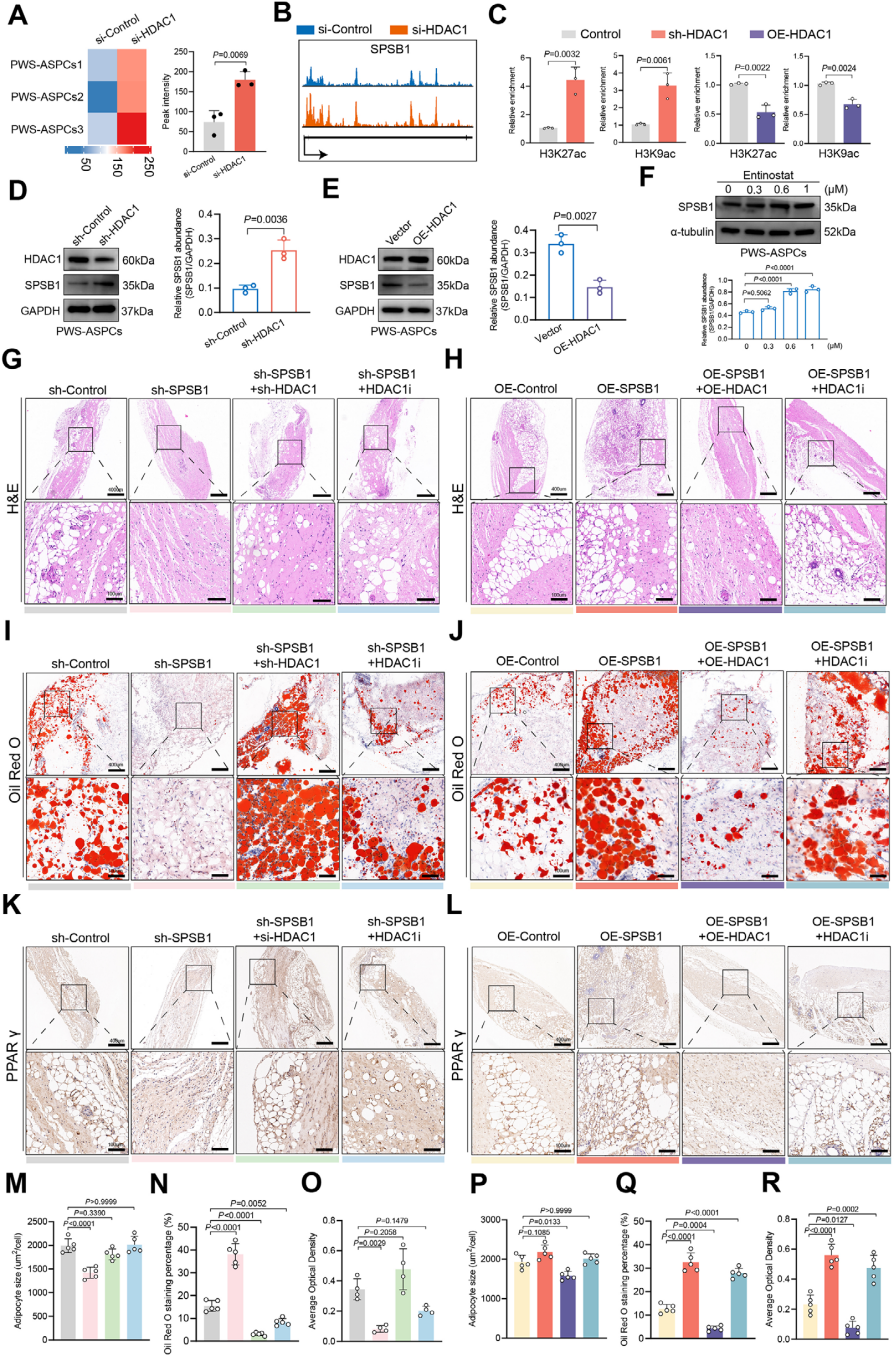
SPSB1‐HDAC1 positive feedback loop regulated adipogenesis. (A): Heatmaps and the peaks intensity quantitative analysis showed the chromatin accessibility of SPSB1 in ATAC‐seq. (B): IGV maps showed the increased ATAC‐seq peaks of SPSB1 promoter. (C): H3K27ac and H3K9ac ChIP‐qPCR validation for SPSB1 promoter regions in PWS‐ASPCs with different interventions. (D): WB and quantitative analysis showed that the SPSB1 and HDAC1 protein levels in PWS‐ASPCs with or without HDAC1 knockdown. (E): WB and quantitative analysis showed that the SPSB1 and HDAC1 protein levels in PWS‐ASPCs with or without HDAC1 overexpression. (F): WB and quantitative analysis showed that the SPSB1 and HDAC1 protein levels in PWS‐ASPCs with different dose of entinostat administration. (G, H): H&E staining of Matrigel implants collected on day 28 and quantification of adipocytes size. Scale bar: up: 400 µm; down: 100 µm. (I, J): Oil Red O staining of Matrigel implants and quantification of Oil red O staining area. Scale bar: up: 400 µm; down: 100 µm. (K, L): The protein level of PPAR γ detected by IHC in the implants of each group. Scale bar: Scale bar: up: 400 µm; down: 100 µm. (M): Statistical analysis of adipocyte size in (G). (N): Statistical analysis of Oil red O staining area in (I). (O): Statistical analysis of PPAR γ intensity in (K). (P): Statistical analysis of adipocyte size in (H). (Q): Statistical analysis of Oil red O staining area in (J). (R): Statistical analysis of PPAR γ intensity in (L). Data were analyzed by unpaired two‐sided Student's t tests (D, E) or one‐way ANOVA (F, M, N, O, P, Q, R), and were presented as mean ± SD with at least three replicate experiments. Original blot can be found in Figure S10.

To determine whether SPSB1 regulated adipogenesis via HDAC1, we performed rescue experiments. We found that knockdown of HDAC1 partially reversed the impairment of adipogenesis caused by SPSB1 knockdown (Figure S9A–C). Conversely, overexpression of HDAC1 counteracted the pro‐adipogenic effect of SPSB1 overexpression (Figure S9D–F). These results indicated that SPSB1 regulated adipogenesis through HDAC1. Prior studies have revealed that PJA2 mediated the degradation of HDAC2, thereby relieving HDAC2‐mediated transcriptional repression of the PJA2 gene and leading to a further increase in PJA2 levels [45]. We sought to investigate whether a similar feedback regulatory mechanism exists in PWS‑ASPCs. ATAC‐seq analysis showed that knocking down HDAC1 increased chromatin accessibility at the SPSB1 gene (Figure 9A,B). ChIP‑qPCR analysis demonstrated that knockdown of HDAC1 increased the enrichment of the active histone marks H3K9ac and H3K27ac at the SPSB1 promoter region, while HDAC1 overexpression produced the opposite effect (Figure 9C). Measurement of SPSB1 mRNA and protein levels following HDAC1 modulation further confirmed that HDAC1 suppressed SPSB1 transcription and consequently downregulated SPSB1 protein expression (Figure 9D, E and Figure S9G–J). Additionally, treatment with the HDAC1 inhibitor Entinostat dose‑dependently upregulated SPSB1 expression (Figure 9F and Figure S9K, L). Collectively, these findings indicated that SPSB1‑mediated downregulation of HDAC1 relieved transcriptional repression of the SPSB1 gene, establishing a positive feedback loop that promotes adipose tissue expansion in PWS.

**FIGURE 10:**
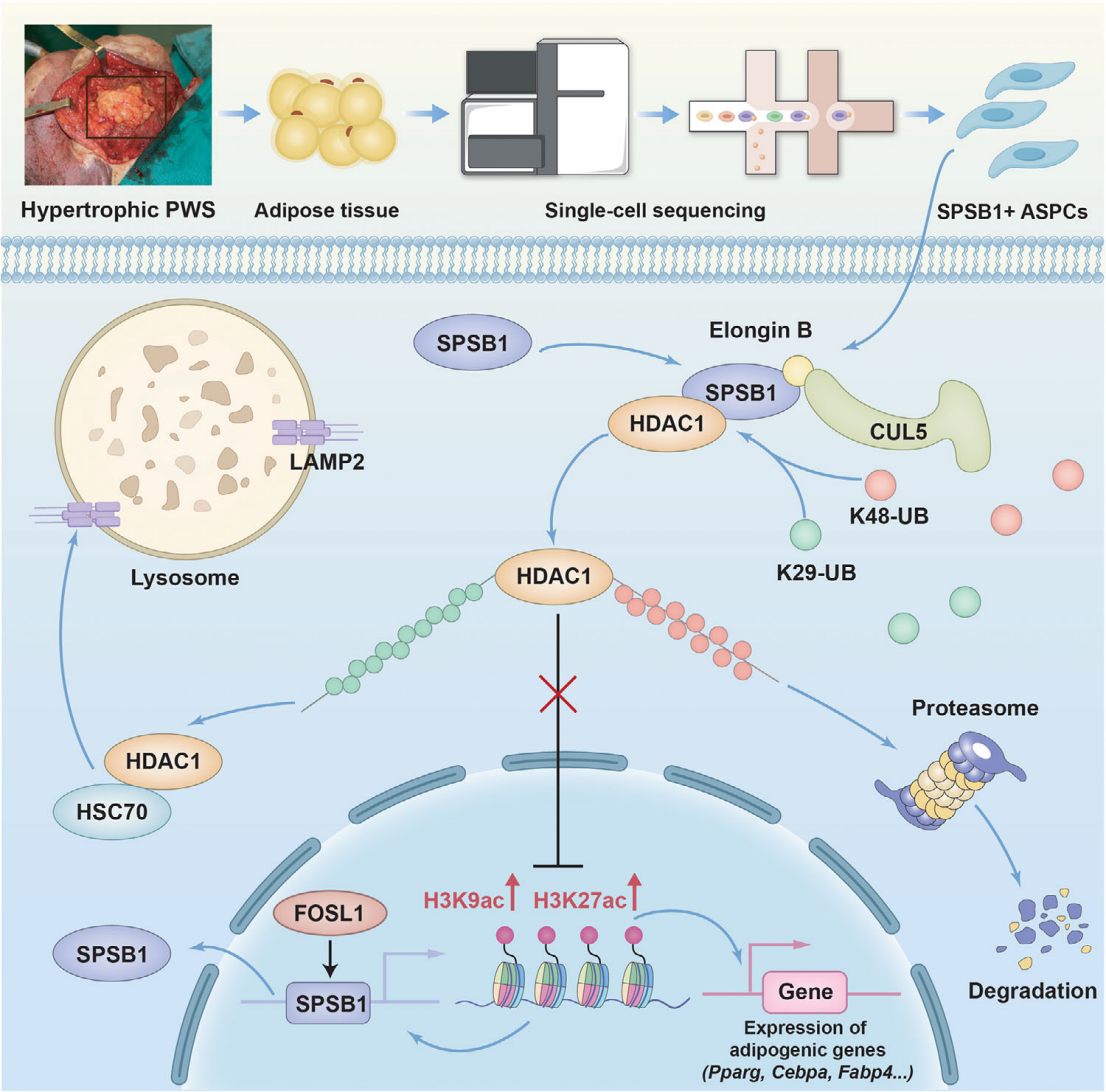
Schematic model of SPSB1‐mediated ubiquitination of HDAC1 in regulation adipogenesis. In PWS‐ASPCs, FOSL1 drives the expression of SPSB1. SPSB1, as part of the ESC complex, further binds to HDAC1 and promotes K29‐linked and K48‐linked polyubiquitination of HDAC1. These modifications facilitate the degradation of HDAC1 through the ALP and UPS pathways, respectively. Reduced HDAC1 levels lead to increased abundance of H3K9ac and H3K27ac at the promoter regions of SPSB1 and adipogenic genes, promoting adipogenic differentiation while further enhancing SPSB1 expression, thereby forming a positive feedback loop.

Finally, we further validated the regulatory role of the SPSB1‐HDAC1 axis in adipogenesis using an in vivo lipoma model. Compared with the group receiving SPSB1 knockdown alone, either HDAC1 knockdown or Entinostat treatment partially rescued the impaired adipogenesis caused by SPSB1 deficiency. This rescue was evidenced by an increase in adipocyte numbers, enhanced lipid accumulation, and elevated PPARγ synthesis (Figure 9G, I, K, and M–O). Conversely, in cells overexpressing SPSB1, overexpression of HDAC1 antagonized the enhanced adipogenic capacity (Figure 9H,J,L,P–R). These results further underscored the critical role of HDAC1 in SPSB1‑mediated promotion of adipogenesis. In summary, our study elucidated a mechanism through which a distinct subpopulation of PWS‑ASPCs with high SPSB1 expression drove the characteristic subcutaneous adipose tissue expansion in PWS. This mechanism involved SPSB1‑mediated degradation of HDAC1, which in turn relieved transcriptional repression on adipogenic genes and promoted adipogenesis (Figure 10).

## 3 Discussion

In this study, we have identified SPSB1 as a critical regulator of subcutaneous adipose hyperplasia in PWS. Through integrative single‐cell transcriptomics, molecular validation, and functional assays, we demonstrated that SPSB1 was highly expressed in a distinct subpopulation of ASPCs from PWS adipose tissue. SPSB1 promoted adipogenesis both in vitro and in vivo, and mechanistically, it interacted with HDAC1 to mediate its degradation via K29‐ and K48‐linked ubiquitination through both the UPS and ALP. The downregulation of HDAC1 enhanced chromatin accessibility and transcriptional activation of adipogenic genes and SPSB1, further establishing a positive feedback loop that sustained SPSB1 expression and drove pathological fat expansion in PWS.

Recent studies have reported that SPSB proteins promoted adipogenic differentiation by facilitating the degradation of FOG‑2 [46]. Our findings reveal a previously unrecognized role of SPSB1 in adipose biology. While SPSB1 has been implicated in cellular growth and cancer progression as an adapter protein in ECS complexes, its function in adipose tissue homeostasis and pathology remains largely unexplored [47, 48]. The identification of FOSL1 as an upstream transcriptional activator of SPSB1 provided further insight into the regulatory network governing SPSB1 expression in PWS‐ASPCs. This aligned with emerging evidence that dysregulated transcription factors in stromal cells contribute to adipose tissue remodeling in various hyperplastic and hypertrophic conditions [49, 50].

The regulation of SPSB1 in broader physiological contexts, including obesity, aging, and pathological fat depot accumulation, remains largely unexplored. Nevertheless, several lines of indirect evidence support the biological plausibility of SPSB1 as a context‐dependent regulator of adipose tissue homeostasis. First, SPSB1 expression is markedly upregulated by pro‐inflammatory cytokines, including TNF‐α, IL‐1β, and IL‐6, via NF‐κB and JAK2/STAT3 signaling pathways in skeletal muscle [51]. Given that chronic low‐grade inflammation is a hallmark of both obesity and aging adipose tissue, it is conceivable that inflammatory in the adipose microenvironment may contribute to the elevated SPSB1 levels [52, 53]. Second, SPSB1, together with SPSB4, participates in the ubiquitin‐mediated degradation of RevErbα, a nuclear receptor that integrates circadian rhythm with hepatic energy metabolism and adipose tissue expansion [54]. This positions SPSB1 at the intersection of circadian disruption and metabolic dysregulation, both of which are prominent features of aging and obesity. We acknowledge that our current study, focused on the mechanistic dissection of the SPSB1‐HDAC1 axis in PWS‐ASPCs, does not address these broader contextual variables. Future investigations employing multi‐ethnic patient cohorts, sex‐stratified analyses, aged animal models, and pharmacological screens will be essential to elucidate whether the SPSB1‐HDAC1 regulatory loop identified here operates universally or is subject to modulation by obesity status, aging, sex, ethnicity, and therapeutic interventions.

Emerging evidence indicates that the ubiquitin syntax decorating HDAC1 is cell‐type‐specific, dictating both the route and rate of its disposal. In vascular smooth‐muscle cells challenged with inorganic phosphate, MDM2 confines to K74 of HDAC1, assembling K48‐linked chains that drive proteasomal degradation [55]. A similar K48 signature is used in autoimmune CD4^+^ T lymphocytes, yet the responsible ligase RNF157 simultaneous targets K74 plus K89 of HDAC1 [56]. The dual proteolytic pathways through which SPSB1 promotes HDAC1 degradation represented a novel mechanistic layer in protein turnover regulation. While K48‐linked ubiquitination typically targets proteins for proteasomal degradation, the involvement of K29‐linked chains in lysosomal targeting via CMA expanded the functional repertoire of ubiquitin signaling in metabolic tissues. Our data suggests that SPSB1 orchestrates a coordinated degradation system to efficiently reduce HDAC1 levels, thereby relieving its repressive effects on adipogenic gene expression. This is consistent with previous reports that HDAC1 inhibits adipogenesis by modulating histone acetylation landscapes, but our study adds mechanistic specificity to this model within the context of PWS [33, 57].

Clinically, our work highlights the potential of targeting the SPSB1‐HDAC1 axis for the treatment of PWS‐associated soft tissue hypertrophy. The partial rescue of aberrant adipogenesis by HDAC1 knockdown or pharmacological inhibition with Entinostat in our experimental models supports the therapeutic relevance of modulating this pathway. Based on the findings of this study that HDAC1 plays a critical role in suppressing adipogenesis and adipose hyperplasia, the development of pharmacological agents that enhance HDAC1 activity represents a promising therapeutic strategy for hypertrophic PWS and related adipose tissue disorders. Yet any strategy that simply boosts global HDAC1 activity must confront its dual effect: while it silences adipogenic loci, the same enzyme displaces acetyl marks from p21 and replication‐origin histones, accelerating G1/S transit [58]. The therapeutic goal is not wholesale activation but contextual rewiring. Moreover, SPSB1 itself could serve as a biomarker for identifying PWS patients at risk of developing adipose hyperplasia or as a target for gene‐based interventions.

Nevertheless, our study has several limitations. The sample size, though sufficient for mechanistic discovery, is relatively small for broad clinical generalizations. Future studies involving larger patient cohorts and longitudinal follow‐up are needed to validate the prognostic value of SPSB1 expression. Additionally, while our murine models recapitulate key aspects of adipogenesis, they do not fully mirror the vascular and inflammatory components of human PWS. Developing patient‐derived organoid or xenograft models may provide more physiologically relevant platforms for preclinical testing.

Looking forward, several questions remain unanswered. Does SPSB1 influence other adipose‐related pathways, such as angiogenesis or fibrosis, which are also relevant to PWS progression? What upstream signals drive FOSL1 activation in PWS‐ASPCs? Furthermore, the interplay between SPSB1‐mediated HDAC1 degradation and other epigenetic modifiers in shaping the adipogenic transcriptome warrants deeper investigation. Integrating multi‐omics approaches across different stages of PWS may uncover dynamic regulatory networks and identify additional therapeutic nodes.

In conclusion, we propose a model in which SPSB1, upregulated by FOSL1 and self‐feedback loop in a unique ASPC subpopulation, promotes HDAC1 degradation via dual ubiquitin‐dependent pathways. This alleviates epigenetic repression of adipogenic genes, enhances lipid accumulation, and establishes a self‐reinforcing loop that drives pathological adipose expansion in facial PWS. Our study not only advances the molecular understanding of PWS‐associated lipohyperplasia but also offers new avenues for targeted therapeutic development.

## 4 Methods

### 4.1 Ethics Approval

This study was conducted in accordance with the Declaration of Helsinki and approved by the Ethics Committee of Shanghai Ninth People's Hospital. The approval number was No.SH9H‐2022‐T215‐1. ASPCs were isolated and collected from facial adipose tissue, with written consent of donors.

### 4.2 ASPCs Isolation, Culture and Identification

Detailed protocol can be found in our previous publication [59].

### 4.3 Adipogenic Induction

In short, we used the Cyagen adipogenic induction kit (Cyagen Biosciences, HUXMD‐90031); the detailed protocol can be found in our previous publication. When required, Induction solution A and B were supplemented with Entinostat (MedChemExpress, HY‐111770). Cells were harvested on day 4 or 8 for subsequent experiments.

### 4.4 Oil Red O Staining

Oil Red O staining was performed according to the instructions of the Modified Oil Red O Staining Kit (Beyotime, C0158S). Quantitative analysis of Oil red O staining area was determined using Image‐Pro Plus 6.0 software.

### 4.5 Mice

All animal procedures were conducted under the approved protocol by the Committee of Animal Care and Use for Research and Education of Shanghai Jiao Tong University School of Medicine. Four weeks old BALB/C nude mice and 6 weeks old C57BL/6J mice were purchased from the Shanghai SLAC Laboratory Animal Co. Both male and female mice were used in a randomized manner. These mice were then individually housed under a 12‐hour light/dark cycle, with access to basic diet and water.

### 4.6 Implant Lipoma Experiment

A total of 1×10^6^ ASPCs were resuspended in 50 µL DPBS and blended 1:1 with Matrigel (Corning, 356234) on ice. The 100 µL cell–Matrigel mixture (1 × 10^6^ cells per mouse) was injected subcutaneously into the dorsal flank of BALB/c nude mice (n = 5 per group). Four weeks after implantation, animals were euthanized by cervical dislocation and the xenografts were collected for histological analysis.

### 4.7 AAV8‐Mediated Selective SPSB1 Overexpression in Adipocytes

AAV8‐FABP4‐SPSB1‐eGFP or AAV8‐FABP4‐Scramble‐eGFP were procured from Genomeditech. A dose of 5×10^11^ virus was resuspended in 150 µL sterile PBS. Six‐week‐old male C57BL/6J mice were anesthetized with isoflurane, after which the viral suspension was delivered by lateral‐tail‐vein injection. Mice were maintained on a normal chow diet throughout the experiment. Six weeks later, SPSB1 overexpression efficiency was evaluated by immunofluorescence and Western blot.

### 4.8 Western Blot

Adipose tissue and ASPC specimens were collected and then homogenized in RIPA lysis buffer (Epizyme, PC101) supplemented with a protease inhibitor cocktail (Epizyme, GRF101) and a phosphatase inhibitor cocktail (Epizyme, GRF102), with all procedures carried out on ice. Following lysis, protein concentrations were determined using a BCA protein assay kit (Epizyme, ZJ102). Equal amounts of total protein were resolved by 10% SDS‑polyacrylamide gel electrophoresis and subsequently transferred onto PVDF membranes (Millipore, ISEQ00010) via electroblotting. To block non‑specific signals, the membranes were treated with a protein‑free rapid blocking buffer (1×) (Epizyme, PS108P) for 10 min at room temperature. Thereafter, membranes were incubated with appropriate primary antibodies overnight at 4°C. After washing with Tris‑buffered saline containing 0.1% Tween 20 (Beyotime, ST1727), the blots were exposed to isotype‑matched secondary antibodies for 45 min at ambient temperature. The primary antibodies used were as follows: anti‐SPSB1 (ThermoFisher, PA5‐70757), anti‐FOSL1 (Proteintech, 12663‐1‐AP), anti‐α‐tubulin (Proteintech, 66031‐1‐Ig), anti‐GAPDH (Epizyme, LF211S), anti‐PPAR γ (Cell Signaling Technology, 2443S), anti‐C/EBP α (Proteintech, 13274‐1‐AP), anti‐FABP 4 (Proteintech, 12802‐1‐AP), anti‐HDAC1 (Proteintech, 80M07L65), anti‐Flag (MERCK, F7425), anti‐Myc (Proteintech, 60003‐2‐Ig), anti‐HA (MERCK, H6908), anti‐Ubiquitin (Proteintech, 10201‐2‐AP), anti‐UB‐K29 (MERCK, SAB5701122), anti‐UB‐K48 (Genscript, A03344‐50), anti‐PSMD4 (Epizyme, 98D48H69), anti‐LAMP2 (Epizyme, 43K44M70), anti‐HSC70 (Proteintech, 10654‐1‐AP), anti‐H3K9ac (HUABIO, HA722132), anti‐H3K27ac (HUABIO, HA500046), anti‐Histone H3 (HUABIO, ET1701‐64). The blot signals were visualized using Pierce ECL Western blot Substrate (Proteintech, PK10003). The abundance of each target protein was quantified via ImageJ software (NIH).

#### 4.8.1 RNA Isolation, Reverse Transcription, and Real‐Time Quantitative PCR (qPCR)

Cultured cells were lysed with TRIzol Reagent (Invitrogen, 15596026CN) to isolate total RNA. Reverse transcription to generate complementary DNA (cDNA) was carried out using the PrimeScript RT Reagent Mix (Takara Bio, RR036A), strictly following the manufacturer's protocol. Quantitative real‑time PCR was subsequently conducted with SYBR Green PCR Master Mix (ThermoFisher, 4309155) on an ABI 7500 real‑time PCR system (Applied Biosystems). Target gene expression levels were normalized to the housekeeping gene GAPDH as well as to corresponding control samples. A complete list of primer sequences was provided in Table S1.

### 4.9 Lc‐Ms/Ms

Detailed protocol can be found in previous publication [60].

#### 4.9.1 Chromatin Immunoprecipitation And Quantitative PCR Validation (ChIP‐qPCR)

PWS‑ASPCs and Im FIL‑ASPCs were treated with 1% formaldehyde for 15 min at ambient temperature to achieve cross‑linking, after which the reaction was terminated by the addition of glycine to a final concentration of 0.125 M. Following cell lysis, nuclei were released via Dounce homogenization in ice‑cold lysis buffer. The resulting chromatin was fragmented by sonication into pieces ranging from 300 to 500 base pairs. An input fraction was prepared by treating an aliquot of the sheared chromatin with RNase A and proteinase K, followed by overnight incubation at 65°C to reverse cross‑links. DNA was then purified through ethanol precipitation, and its concentration was measured using a NanoDrop spectrophotometer to estimate total chromatin recovery. For immunoprecipitation, 30 µg of chromatin was first pre‑cleared with protein A‑agarose beads (Invitrogen, 78610) and subsequently incubated with antibodies targeting H3K9ac and H3K27ac. The resulting immune complexes were collected, washed thoroughly, and eluted in a buffer containing SDS. After treatment with RNase and proteinase K, the eluates were incubated overnight at 65°C to reverse cross‑links. ChIP‑enriched DNA was recovered by phenol‑chloroform extraction followed by ethanol precipitation. Enrichment levels were assessed in triplicate qPCR reactions using SYBR Green Supermix (Bio‑Rad) at selected genomic loci, with values normalized to the corresponding input DNA. Primer sequences used in this study were provided in Table S1.

#### 4.9.2 Proximity Ligation Assay (PLA)

PWS‑ASPCs and Im FIL‑ASPCs were seeded onto confocal‑compatible dishes and allowed to adhere overnight. Following a 24‑hour incubation period, cell monolayers were fixed with 4% paraformaldehyde for 1 h at 37°C, followed by permeabilization and blocking steps. A mixture of primary antibodies recognizing SPSB1 and HDAC1 was applied and incubated overnight at 4°C. On the next day, all subsequent procedures—including probe hybridization, ligation, and rolling‑circle amplification—were performed strictly according to the NavinciFlex Cell MR protocol (Navinci, Atto647N). Nuclear counterstaining was carried out using DAPI, and the resulting proximity ligation signals were visualized using a Thunder Imager high‑speed inverted fluorescence microscope (Leica).

### 4.10 Isolation of Lysosomal Proteins

Lysosomal fractions were isolated according to the kit manual (Thermo). In brief, 5 × 10^7^ pancreatic cancer cells were detached with the supplied reagents, sonicated, and the homogenate subjected to ultracentrifugation at 145 000 × g, 4°C. The resulting pellet was resuspended in Laemmli buffer and analysed by immunoblotting.

### 4.11 Protein Half‐life Analysis

ASPCs cells were treated with the protein synthesis inhibitor CHX (C7698, Sigma) and other degradation inhibitor for the indicated time courses. Cells were harvested at the indicated time post‐CHX treatment for immunoblot analysis using the indicated antibodies.

### 4.12 Plasmid and siRNA Transfection

Plasmids containing Flag‐SPSB1, Myc‐HDAC1 and HA‐UB were obtained from Zuorun Bio (Shanghai, China). Small silencing RNAs (si‐HSC70, si‐HDAC1) were purchased from Genomeditech Bio (Shanghai, China). Plasmids or siRNAs were transfected into 60%‐70% confluent ASPCs with Lipo8000 reagent (Beyotime, C0533). Medium was changed to basal medium after 4–6 h transfection before the following experiments.

### 4.13 Immunohistochemistry

For immunohistochemistry staining, the deparaffinized and hydrated histological slides of adipose tissue were blocked by 5% bovine serum albumin for 30 min at room temperature and incubated overnight (4°C) with primary antibodies. Next, the slides were washed with TBST (G0004; Servicebio), followed by the incubation with secondary antibodies, HRP‐conjugated goat anti‐rabbit IgG (GB23303; Servicebio).

### 4.14 Immunofluorescent Staining

Sections were first deparaffinized, followed by antigen retrieval using either EDTA (pH = 9.0) or sodium citrate (pH = 6.0) solutions, applying high pressure for 2 min. Afterward, the sections were incubated with 10% goat serum at ambient temperature for 1 h to block non‐specific binding, then treated with primary antibodies overnight at 4°C. The sections underwent three washes with Phosphate buffered saline before being incubated with Alexa Fluor 488 goat anti‐rabbit (Invitrogen, A‐11008) and Alexa Fluor 594 goat anti‐mouse (Abcam, ab150116) secondary antibodies for 40 min at room temperature. Finally, sections were coverslipped using a 4′,6‐diamidino‐2‐phenylindole (DAPI) based mounting medium (ZLI‐9557, ZSGB‐BIO).

### 4.15 Lentivirus Packaging

We prepared lentivirus to knockdown or overexpress the expression of SPSB1 and HDAC1. The lentivirus was obtained from Zuorun Biotech. For transduction, ASPCs were incubated with virus‐containing supernatant (MOI = 10) in the presence of 10 µL polybrene. After 10 h, infected cells were selected with puromycin supernatant.

### 4.16 RNA Sequencing and Data Analysis

RNA sequencing was conducted by Genefund Biotech (Shanghai, China). In brief, homogenized ASPCs were subjected to total RNA extraction using the RNeasy Mini Kit (QIAGEN, 74004). RNA concentration was assessed with a NanoDrop 2000 spectrophotometer, and sample integrity was evaluated on an Agilent 2100 Bioanalyzer; only specimens displaying an RNA integrity number (RIN) of 8.0 or higher were considered suitable for downstream processing. For each biological replicate, 3 µg of total RNA served as input for library preparation, which was carried out using the VAHTS Universal V6 RNA‑seq Library Prep Kit according to the manufacturer's instructions. The resulting libraries were sequenced on a NovaSeq 6000 platform to generate paired‑end reads of 150 bp in length. Transcript abundances at the gene level were quantified as FPKM values using featureCounts (version 2.0.1). Differential expression analysis was performed with DESeq2 (version 1.30.1); genes meeting the criteria of an adjusted p‑value below 0.05 and an absolute log2 fold change greater than 1 (or greater than 0.5 where explicitly indicated) were retained for subsequent analyses. Volcano plots were generated using the ggplot2 package in R, while heatmaps were constructed with GENE‑E software (Broad Institute).

### 4.17 ATAC Sequencing and Data Analysis

ATAC‑seq analyses were carried out by Genefund Biotech (Shanghai, China). Briefly, ASPCs underwent cell lysis and subsequent nuclear isolation according to a standard protocol. Transposition reactions and Illumina‑compatible library construction were performed using the TruePrep DNA Library Prep Kit V2 for Illumina (Vazyme, TD501) following the manufacturer's recommendations. Following amplification and quality assessment, the resulting libraries were sequenced on a NovaSeq 6000 platform, yielding 150‑bp paired‑end reads. Raw sequencing reads were processed with fastp (version 0.23.1) to eliminate adapter sequences and low‑quality bases. Sambamba (version 0.7.1) was employed for SAM/BAM file conversion and removal of duplicate reads. Read density profiles centered on transcription start sites (TSS) were visualized using DeepTools (version 2.4.1). Genomic distribution of sequencing signals was evaluated with RSeQC (version 2.6). Motif enrichment analysis was conducted using Homer (version 4.10), while identification of differentially accessible peaks was performed with csaw (version 1.24.3).

### 4.18 Molecular‐Dynamics (MD) Simulations

The detailed protocol can be found in our prior publication [61].

### 4.19 Single Cell Sequencing Analysis

Single cell sequencing was performed by Singleron Biotechnologies. The detailed analytical workflow was described in our previously publication [32].

### 4.20 Quantification and Statistical Analysis

GraphPad Prism (v 8.0) was used for statistical analyses. Two‐tailed Student's t test was utilized for comparison between the two groups. One‐way analysis of variance (ANOVA) coupled with the Bonferroni's post hoc test was used for comparisons among three or more groups. Data were presented as mean ± standard deviation (SD). *P* < 0.05 was considered of statistical significance.

### 4.21 Declarations

#### 4.21.1 Ethics Approval and Consent to Participate

The study was approved by the Ethics Board of Shanghai Ninth Hospital, Shanghai Jiaotong University of Medicine (SH9H‐2022‐T215‐1). Written informed consent forms were signed by patients or patients’ parents whose facial adipose sample were collected in this study. Title of the approved project: Treatment and mechanism of Pl3K/mTOR dual‐target inhibitor (WX390) on PlK3CA‐related overgrowth spectrum (PROS). Date of approval: 06, October, 2022. All animal procedures were performed following protocols approved by the Institutional Animal Care and Use Committee (IACUC) of the Shanghai Jiao Tong University School of Medicine (SH9H‐2022‐A916‐1). Title of the approved project: Etiological Research on Limb Developmental Defects and Construction of a Precision Prevention and Treatment System: Research on the Pathogenesis and Targeted Therapy of Limb Proliferative Diseases Caused by Somatic Activating Mutations. Date of approval: 11, November, 2022.

## Author Contributions

**H.C**.: Conceptualization, methodology, software, investigation, formal analysis, Writing – original draft. **B.S**.: Data Curation, writing – original draft. Y.Q.: Resources, supervision, writing – review & editing. **C.H**.: Software, validation, writing – review & editing. **X.L**.: Visualization, writing – review & editing. **W.G**.: Writing – review & editing.

## Funding

This research was supported by Top Priority Research Center of Shanghai—Plastic Surgery Research Center, Shanghai (2023ZZ02023), Clinical Safety and Efficacy of 2940 nm Fractional Erbium Laser in Treating Periorbital Skin Fine Lines and Laxity (JYHX2025017), a Study on the Efficacy and Safety of Fractional CO2 Laser Ablative Therapy at Different Densities for Periorbital Static Wrinkles in Chinese Population (JYHX2025043), and the use of high‐frequency microneedle technology and microfocused ultrasound alone or in combination for faciai rejuvenation: a prospective, randomized, half face, comparative clinical trial (JYHX2024008).

## Consent

The consent for publication was acquired from patients’ parents. All authors confirm their consent for publication.

## Conflicts of Interest

The authors declare no conflicts of interest.

## Supporting information




**Supporting File 1**: advs76699‐sup‐0001‐SuppMat.docx.

**Supporting File 2**: advs76699‐sup‐0002‐TableS1.docx.

## Data Availability

The datasets used and/or analyzed during the current study are available in NCBI's Gene Expression Omnibus (GEO) or China National Center for Bioinformation/Beijing Institute of Genomics, Chinese Academy of Sciences, and are accessible through accession number GSA‐Human:HRA015590 (scRNA‐seq in Figures 1, 2), GSA‐Human: HRA014905 (RNA‐seq in Figure 8), and GSA‐Human: HRA014913 (ATAC‐seq in Figure 8).
