# Supplementary material for: SPSB1 Promotes Subcutaneous Adipose Hyperplasia in Facial Port‐Wine Stains by Controlling HDAC1 Degradation and Stability Through Two Distinct Proteolytic Pathways
Source: Adv Sci (Weinh). 2026 Jul 20;13(57):e76699. doi: 10.1002/advs.76699 (PMC13383696; doi:10.1002/advs.76699)
Supplement: Supplementary file 1 [file ADVS-13-e76699-s001.docx]

**Table S1. Primers used in this study**

**Primer for qPCR:**

| **Gene/RNA** | **Forward primer (5'→3')** | **Reverse primer (5'→3')** |
| --- | --- | --- |
| GAPDH | CATCATCCCTGCCTCTACTGG | GTGGGTGTCGCTGTTGAAGTC |
| SPSB1 | AGTACATGGGAGTGGCTTTTC | ACAAATCCATGAGCGGCAG |
| HDAC1 | GCGAGCAAGATGGCGCAGACG | AGCATTGGCTTTGTGAGGGCGAT |
|  |  |  |

**Primer for ChIP-qPCR**

| **Gene/RNA** | **Forward primer (5'→3')** | **Reverse primer (5'→3')** |
| --- | --- | --- |
| PPARG | TCCTGGGAGCCTAACTGAG | CTTCCACCAAGGGACCTGAG |
| CEBPA | CTCCTCCTGCCTGCCCTA | GTGCAGCCTCGGGATACTC |
| FABP4 | TGGTTCTCCCTGGCAAATAG | CATTAAGCTGTCAAAACAGGAATG |
