# Supplementary material for: SPSB1 Promotes Subcutaneous Adipose Hyperplasia in Facial Port‐Wine Stains by Controlling HDAC1 Degradation and Stability Through Two Distinct Proteolytic Pathways
Source: Adv Sci (Weinh). 2026 Jul 20;13(57):e76699. doi: 10.1002/advs.76699 (PMC13383696; doi:10.1002/advs.76699)
Supplement: Supplementary file 2 [file ADVS-13-e76699-s002.docx]

**Supplementary figures**

**Figure S1: Characteristics of ASPCs.** A: Volcano plot showing the differences in the expression of genes involved in fat cell differentiation in PWS-ASPCs and CON- ASPCs. B-C: Dimensionality reduction plot displaying the distribution of CytoTRACE scores (B) and Box plot (C) comparing CytoTRACE scores across these nine ASPCs subpopulations.

**Figure S2: FOSL1 promoted SPSB1 expression.** A: ChIP-qPCR analysis showing significant enrichment of FOSL1 at the promoter region of SPSB1. B: Luciferase reporter assay suggesting that FOSL1 enhanced the transcriptional activity of the wild-type SPSB1 promoter relative to a mutant type. C: qPCR analysis of SPSB1 mRNA level in PWS-ASPCs with or without FOSL1 knockdown. D: qPCR analysis of SPSB1 mRNA level in PWS-ASPCs with or without FOSL1 overexpression. E-F: Western blot and quantitative (F) analysis of SPSB1 protein level in Im FIL-ASPCs with FOSL1 knockdown or overexpression. Data were analyzed by unpaired two-sided Student’s t tests (A, C, D, F) and were presented as mean ± SD with three replicate experiments. Original blot can be found in Figure S10.

**Figure S3:** **AAV8-mediated SPSB1 adipocytic overexpression promoted adipose hyperplasia.** A: WB showing SPSB1 expression in Im FIL-ASPCs upon SPSB1 knockdown. B: WB analysis showed that the protein levels of PPAR γ, C/EBP α, and FABP 4 upon SPSB1 knockdown compared with the wild type at day 3 of adipogenesis. C: WB showing SPSB1 expression in Im FIL-ASPCs upon SPSB1 overexpression. D: WB analysis showed that the protein levels of PPAR γ, C/EBP α, and FABP 4 upon SPSB1 overexpression compared with the wild type at day 3 of adipogenesis. E: Schematic diagram of the experimental process in mice (n=5 per group). F: Representative image of eGFP expression 6 weeks post injection of AAV. Scale bar: 100 μm. G: IF staining and quantitative analysis showed a significant increase in SPSB1 protein expression in adipocytes of the SPSB1 group compared with Scramble. Scale bar: 100 μm. H: SPSB1 expression was analyzed by WB in adipose tissue of SPSB1 group and Scramble group. I: Overview of the body size of the mice from different groups. J: Body weight curve. K: Serum triglyceride (TG), total cholesterol (TC), high-density lipoprotein cholesterol (HDL-C) and low-density lipoprotein cholesterol (LDL-C) levels in different groups of mice. L: Pictures of subcutaneous and visceral adipose tissue in different groups. M: Weights and relative weights of subcutaneous and visceral adipose tissue. N: H&E staining showed the size of subcutaneous adipocytes from different groups. Scale bar: 100μm. O: H&E staining showed the size of visceral adipocytes from different groups. Scale bar: 100μm. P: IF staining showed a significant increase in PPAR γ protein expression in adipocytes of the SPSB1 group compared with Scramble. Scale bar: 100 μm. Data were analyzed by unpaired two-sided Student’s t tests (C, D, G, H, K, M, N, O, P) or one-way ANOVA (A, B), and were presented as mean ± SD with at least three replicate experiments. Original blot can be found in Figure S10.

**Figure S4:** **Molecular dynamics simulation analysis of the SPSB1-HDAC1 complex.** The RMSD-versus-time curve reflects the overall stability of the protein-protein complex during the 10 ns molecular dynamics simulation. B: The RMSF plot depicts the flexibility changes of the SPSB1 protein during the molecular dynamics simulation. C: The RMSF-versus-residue curve illustrates the residue-specific flexibility profile of HDAC1 throughout the molecular dynamics simulation. D: Evolution of the radius of gyration (Rg) for the system during the 0-10 ns molecular dynamics trajectory (time in ps). E: Variation of the solvent-accessible surface area (SASA) of the system throughout the 10-ns molecular dynamics simulation. F: Number of interfacial hydrogen bonds (black line) and donor-acceptor pairs within 0.35 nm (red line) during the 10-ns simulation. G: Time evolution of the minimum inter-atomic distance between SPSB1 and HDAC1 during the molecular dynamics simulation. H: Free-energy landscape (FEL) plotted with RMSD (conformational deviation) on the x-axis and Rg (radius of gyration, reflecting overall compactness) on the y-axis; colors encoded Gibbs free energy, with blue indicating low-energy (most stable) states and red high-energy (unstable) states. I: Three-dimensional free-energy landscape (Gibbs energy) derived from molecular-dynamics simulations. The horizontal axis (PC1) denoted RMSD, reporting conformational deviation; the vertical axis (PC2) corresponded to Rg, reflecting overall compactness; the height axis represented Gibbs free energy. Color and elevation jointly encode energy magnitude: blue depressions marked the lowest-energy, most stable conformations, whereas red plateaus indicated high-energy, unstable regions.

**Figure S5:** **The physical interaction between SPSB1 and HDAC1.** A: Detailed hydrogen bonding sites between amino acids in SPSB1 and HDAC1. B: IP and western blot assay indicating the interactions between FLAG-tagged truncated SPSB1 and Myc-tagged HDAC1 proteins in HEK293T cells. Cell extracts were IP with an anti-Myc antibody. C: IP and western blot assay indicating the interactions between FLAG-tagged SPSB1 and Myc-tagged truncated HDAC1 proteins in HEK293T cells. Cell extracts were IP with an anti-Flag antibody. Original blot can be found in Figure S10.

**Figure S6: SPSB1 promoted HDAC1 degradation.** A: WB and quantitative analysis showed that the protein levels of HDAC1 upon SPSB1 knockdown in Im FIL-ASPCs. B: qPCR analysis showed that the mRNA levels of HDAC1 upon SPSB1 knockdown in Im FIL-ASPCs. C: WB and quantitative analysis showed that the protein levels of HDAC1 upon SPSB1 overexpression in Im FIL-ASPCs. D: qPCR analysis showed that the mRNA levels of HDAC1 upon SPSB1 overexpression in Im FIL-ASPCs. E: Im FIL-ASPCs were transfected with plasmids encoding Myc-tagged HDAC1, along with a plasmid encoding Flag-tagged wild-type SPSB1 or SPSB1 mutants. Cell lysates were analyzed by western blot with indicated antibodies. F: Im FIL-ASPCs were transfected with plasmids encoding Myc-tagged HDAC1, along with a plasmid encoding Flag-tagged wild-type SPSB1 or SPSB1 mutants. Cell lysates were analyzed by qPCR. G-I: Im FIL-ASPCs were transfected with the indicated lentivirus. After transfection, cells were treated with 100 μg/mL CHX and collected for immunoblot analysis at the indicated time points. J-K: WB analysis of whole cellular lysates and IPs derived from lysates of Im FIL-ASPCs using indicated K29-UB or K48-UB antibodies. Cells were treated with 20 µM MG132 for 4 h before harvesting. L-M: WB analysis of whole cellular lysates and anti-HDAC1 IPs derived from Im FIL-ASPCs stably expressing sh-SPSB1. Cells were treated with 20 µM MG132 for 4 h before harvesting. N: ECS complexes mediated HDAC1 ubiquitylation. Immunoprecipitated endogenous HDAC1 from control knockdown, Cul5 knockdown or ElonginB/Cul5 double knockdown HEK293T cells was immunoblotted with anti-Ub antibody. Data were analyzed by unpaired two-sided Student’s t tests (C, D, F) or one-way ANOVA (A, B, E), and were presented as mean ± SD with at least three replicate experiments. Original blot can be found in Figure S10.

**Figure S7: HSC70 interacted with HDAC1.** A: Detailed information of KFERQ-like motifs in human HDAC1. B-C: The exogenous interaction between HDAC1 and HSC70 was detected by Co-IP and western blotting assays in Im FIL-ASPCs (B) and PWS-ASPCs (C). D-E: Co-IP assays and quantitative analysis (E) were performed to determine whether Myc-HDAC1 interacted with HA-HSC70 in HEK293Ts and Im FIL-ASPCs transfected with Myc-HDAC K361R. F: Myc-HDAC1 and Flag-HSC70, Flag-HOP, or Flag-LAMP2 were detected by immunofluorescence staining in Im FIL-ASPCs with HDAC1 K361R mutant (left). The co-localization analysis was performed by Image J (right). G-J: Western blot and quantitative (H, J) analysis of PWS-ASPCs and Im FIL-ASPCs transfected with HSC70 siRNAs after CQ treatment. K: Mechanistic model illustrating that K29-UB of HDAC1 enhancing its interaction with HSC70 and subsequent chaperone-mediated delivery to lysosomes for degradation. Data were analyzed by unpaired two-sided Student’s t tests (E, H, J) and were presented as mean ± SD with at least three replicate experiments. Original blot can be found in Figure S10.

**Figure S8:** **HDAC1 regulated chromatin opening.** A: WB and quantitative analysis for the expression of H3K27ac and H3K9ac in Im FIL-ASPCs with or without SPSB1 knockdown. B: WB and quantitative analysis for the expression of H3K27ac and H3K9ac in Im FIL-ASPCs treated with entinostat. C-D: WB and quantitative analysis for the expression of H3K27ac and H3K9ac in PWS-ASPCs (C) and Im FIL-ASPCs (D) with or without SPSB1 knockdown. E: H3K27ac ChIP-qPCR validation for selected gene promoter regions (PPARG, CEBPA, FABP4) in PWS-ASPCs and Im FIL-ASPCs with different interventions. F: H3K9ac ChIP-qPCR validation for selected gene promoter regions (PPARG, CEBPA, FABP4) in PWS-ASPCs and Im FIL-ASPCs with different interventions. G: The expression of HDAC1 and the adipogenic genes was measured by the Chipbase database. H: GO analysis of the differentially accessible regions peaks at candidate target genes. I: KEGG analysis of the differentially accessible regions peaks at candidate target genes. Data were analyzed by unpaired two-sided Student’s t tests (A, B, C, D, E, F) and were presented as mean ± SD with at least three replicate experiments. Original blot can be found in Figure S10.

**Figure S9: SPSB1-HDAC1 positive feedback loop regulated adipogenesis.** A: Oil red O staining presented the lipid accumulation in Im FIL-ASPCs with indicated interference after adipogenic induction for 8 days. B-C: WB and quantitative (C) analysis showed the PPAR γ and C/EBP α expression in Im FIL-ASPCs with indicated interference after adipogenic induction for 3 days. D: Oil red O staining presented the lipid accumulation in Im FIL-ASPCs with indicated interference after adipogenic induction for 8 days. E-F: WB and quantitative (E) analysis showed the PPAR γ and C/EBP α expression in Im FIL-ASPCs with indicated interference after adipogenic induction for 3 days. G: WB and quantitative analysis showed that the SPSB1 and HDAC1 protein levels in Im FIL-ASPCs with or without HDAC1 knockdown. H: WB and quantitative analysis showed that the SPSB1 and HDAC1 protein levels in Im FIL-ASPCs with or without HDAC1 overexpression. I: qPCR analysis showed the mRNA expression of SPSB1 after HDAC1 knockdown or overexpression in PWS-ASPCs. J: qPCR analysis showed the mRNA expression of SPSB1 after HDAC1 knockdown or overexpression in Im FIL-ASPCs. K: WB and quantitative analysis showed that the SPSB1 and HDAC1 protein levels in Im FIL-ASPCs with different dose of entinostat administration. L: qPCR analysis showed the mRNA expression of SPSB1 after entinostat administration in PWS-ASPCs and Im FIL-ASPCs. Data were analyzed by unpaired two-sided Student’s t tests (G, H, I, J) or one-way ANOVA (A, C, D, E, K, L), and were presented as mean ± SD with at least three replicate experiments. Original blot can be found in Figure S10.

**Figure S10: Original Western blot images.**
